# CDK4/6 Inhibition Induces Senescence and Enhances Radiation Response by Disabling DNA Damage Repair in Oral Cavity Squamous Cell Carcinoma

**DOI:** 10.3390/cancers15072005

**Published:** 2023-03-28

**Authors:** Nitisha Shrivastava, Claudia Gutierrez Chavez, Daniel Li, Vikas Mehta, Carlos Thomas, Cory D. Fulcher, Nicole Kawachi, Danielle M. Bottalico, Michael B. Prystowsky, Indranil Basu, Chandan Guha, Thomas J. Ow

**Affiliations:** 1 Department of Pathology, Montefiore Medical Center, Albert Einstein College of Medicine, Bronx, NY 10461, USA; nitisha.shrivastava@einsteinmed.edu (N.S.);; 2 Department of Radiation Oncology, Montefiore Medical Center, Albert Einstein College of Medicine, Bronx, NY 10461, USA; clgutich@gmail.com; 3 Yale School of Medicine, Yale University, New Haven, CT 06510, USA; 4 Department of Thoracic Surgery, Memorial Sloan Kettering Cancer Center, New York, NY 10065, USA; 5 Department of Otolaryngology-Head and Neck Surgery, Montefiore Medical Center, Albert Einstein College of Medicine, Bronx, NY 10461, USA; 6 Cleveland Clinic, 9500 Euclid Avenue, Cleveland, OH 44195, USA; 7 Office of Grant Support, Albert Einstein College of Medicine, Bronx, NY 10461, USA; 8 Urology, Montefiore Medical Center, Albert Einstein College of Medicine, Bronx, NY 10461, USA; 9 Institute for Onco-Physics, Albert Einstein College of Medicine, Bronx, NY 10461, USA

**Keywords:** head and neck squamous cell carcinoma (HNSCC), oral cavity, organoids, PD-0332991 (palbociclib), senescence, CDK4, CDK6, radiosensitizing, DNA repair

## Abstract

**Simple Summary:**

Human papilloma virus–negative (HPV(−)) oral cavity squamous cell carcinoma (OCSCC) is the leading cause of mortality amongst head and neck cancers. Radiation resistance remains a prime cause of treatment failure in OCSCC. Overall, failure to cure locally advanced OCSCC remains a formidable challenge. The aim of our study was to exploit the hyperactive CDK4/6 axis in HPV(−) OCSCC by targeting it with the p16 mimetic palbociclib and to assess the resulting effect on susceptibility to radiation. Our study demonstrates that both homologous recombination (HR) and *Non-homologous end joining pathway* (NHEJ), two critical DNA damage repair pathways, are compromised after palbociclib-induced senescence in OCSCC cells, leading to enhance radiation sensitivity. Our findings provide important insight towards a promising treatment paradigm in OCSCC.

**Abstract:**

Purpose: HPV(−) OCSCC resists radiation treatment. The *CDKN2A* gene, encoding p16INK4A, is commonly disrupted in OCSCC. p16 inhibits CDK4/CDK6, leading to cell cycle arrest, but the biological sequelae of CDK4/6 inhibition in OCSCC remains understudied. This study examines whether inhibition of CDK4/6 enhances radiation response in OCSCC. Methods: MTT assays were performed in OCSCC cell lines HN5 and CAL27 following treatment with palbociclib. Clonogenic survival and synergy were analyzed after radiation (RT-2 or 4Gy), palbociclib (P) (0.5 µM or 1 µM), or concurrent combination treatment (P+RT). DNA damage/repair and senescence were examined. CDK4/6 were targeted via siRNA to corroborate P+RT effects. Three-dimensional immortalized spheroids and organoids derived from patient tumors (conditionally reprogrammed OCSCC CR-06 and CR-18) were established to further examine and validate responses to P+RT. Results: P+RT demonstrated reduced viability and synergy, increased β-gal expression (~95%), and ~two-fold higher γH2AX. Rad51 and Ku80 were reduced after P+RT, indicating impairment of *both* HR and NHEJ. siCDK4/6 increased senescence with radiation. Spheroids showed reduced proliferation and size with P+RT. CR-06 and CR-18 further demonstrated three-fold reduced proliferation and organoids size with P+RT. Conclusion: Targeting CDK4/6 can lead to improved efficacy when combined with radiation in OCSCC by inducing senescence and inhibiting DNA damage repair.

## 1. Introduction

Outcomes among patients with advanced (stage III or IV) oral cavity squamous cell carcinoma (OCSCC) are poor, with 30% developing locoregional failure and 25% at risk for distant failure [1,2]. Radiation therapy (RT) is a cornerstone of all curative approaches for advanced OCSCC, while cisplatin-based chemotherapy and immune-checkpoint inhibitors are used to palliate unresectable and metastatic disease [3,4]. Radioresistance in OCSCC remains a significant problem [5]. Despite robust understanding of the genomic and molecular drivers of OCSCC [6,7,8,9], no current treatments are based on the genomic background underlying this disease, and no approaches target specific mechanisms of RT resistance. 

Alterations of cell cycle signaling are observed in both HPV (+) [10,11] and HPV(−) HNSCC (head and neck squamous cell carcinoma) [12,13]. HPV(−) OCSCC is driven by loss of key tumor suppressors, importantly p16INK4a (p16). *CDKN2A* encodes p16 and is affected in up to 80% of HNSCC; it is often deleted, hyper-methylated, or, much more rarely, mutated. p16 inhibits cyclin-dependent kinases CDK4 and CDK6 (CDK4/6). CDK4/6 activate multiple transcriptional regulators that collectively promote passage through the G1/S checkpoint, suppress reactive oxygen species, and avoid senescence [14]. The CDKN2A gene is predominantly intact and p16 is functional and over expressed in HPV(+) HNSCC [11,15], likely due to lack of negative feedback as downstream Rb is disrupted by the HPV oncoprotein E7. In HPV(−) HNSCC, the *CDKN2A* gene is disrupted early in the carcinogenesis process [16]. Studies suggest that functional p16 may contribute to improved RT responses in HPV(+) tumors [15]. Improving radiation response is particularly important with HPV(−) HNSCC because of high locoregional failure despite aggressive RT [17,18,19]. We therefore postulated that re-establishing CDK4/6 inhibition in HPV(−) OCSCC could enhance the effects of radiation.

The selective CDK4/6 inhibitor palbociclib is approved by the Federal Drug Administration (FDA). This anti-proliferative, cytostatic agent is effective in eliminating cancer cells when used with other drugs [20,21]. While palbociclib has been extensively studied in solid tumors, there is little data in HNSCC. Palbociclib has been shown to induce DNA damage and inhibits homologous recombination (HR) DNA damage repair by inducing cellular senescence and apoptosis in OCSCC [22]. HPV(−) HNSCC cells are highly sensitive to palbociclib, and it has been shown to consistently induce senescence [23]. Palbociclib augments the effects of RT by suppressing DNA damage repair (DDR) and inducing apoptosis in nasopharyngeal carcinoma cells [24]. Despite early promising studies, the data that illustrate the potential efficacy of palbociclib when combined with radiation remains limited in HPV(−) OCSCC. Thus far, only one study has shown palbociclib increases radiosensitivity in HPV(−) HNSCC [25]. The underlying mechanisms of the potential synergy between inhibition of CDK4/6 and radiation in OCSCC remain largely unknown.

The differential induction of senescence is a primary contributor to altered radiation sensitivity in cancers [26]. Senescence is a state of cell-cycle arrest which can serve as a barrier to tumorigenesis [27], can prevent malignant progression [28], and which must be overcome in order for a cancer cell to achieve immortalization [29]. RT-induced senescence has been shown to decrease self-renewal capacity, clonogenicity, and long-term re-population [30]. The senescent state also alters cell response to DNA damaging treatments, such as RT. p16/Rb pathway alterations are required for cancer cells to bypass senescence [31]. Thus, re-establishing senescence pathways in the setting of radiation injury may overcome key hallmarks of cancer cell survival.

CDK4/6 inhibitors may thus represent potentially effective and novel options as radiosensitizers in the OCSCC treatment settings. In this study, we hypothesized that combining palbociclib and radiation would lead to more pronounced senescence, reduced DNA damage repair, and increased cell death in comparison to either modality alone. 

## 2. Materials and Methods

### 2.1. Cell Culture

HN5, CAL27 and HOK16B was obtained from an institutional repository at MD Anderson (courtesy of Jeffrey N. Myers, MD, PhD) with agreements and permissions from the original sources [32]. Swiss 3T3J2 fibroblast cells were obtained from Georgetown University Medical center (courtesy of Richard Schlegel, MD, PhD). CAL27, HOK16B, and 3T3J2 were obtained from all cells (except primary oral keratinocyte) were maintained in Dulbecco’s Modified Eagle’s Medium (DMEM), supplemented with 10% FBS, non-essential amino acids, sodium pyruvate, and 1% penicillin/streptomycin. The immortalized human oral keratinocyte HOK16B were maintained in the serum free keratinocyte growth medium (PCS-200-040) obtained from American Type Culture Collection (ATCC, Manassas, VA, USA). The cells were incubated at 37 °C in 5% CO_2_. All cell lines were STR validated at the time of experiments.

### 2.2. Palbociclib

Palbociclib (PD 0332991) was obtained from Selleck Chemicals, Houston, TX, USA.

### 2.3. MTT Assay

Palbociclib cytotoxicity was measured with MTT; 1000 cells/well were seeded in 96-well plates and treated with palbociclib for 24 h post-seeding (0.01–1 µM). Medium was removed following 72 h and MTT reagent was added (1 mg/mL). Plates were incubated for 3 h, supernatant was decanted, and formazan crystals were dissolved in 150 µL of DMSO. Absorbance (570 nm) was read using ELISA plate reader (BioRad, Hercules, CA, USA). 

### 2.4. Gamma Irradiator 

Gamma radiation (0–6 Gray (Gy) ^137^Cesium irradiator) was administered on a rotating turnstile (7 rpm for uniform dosing) at 100–200 cGy/min. 

### 2.5. Culture Treatment

Briefly, 24 h post-seeding, cells were treated with or without palbociclib (0.5 or 1 µM) added immediately after RT (0, 2 or 4 Gy). Unless otherwise stated, cells were maintained in treated conditions for 72 h prior to any of the mechanistic and colorimetric staining studies.

### 2.6. Clonogenic Survival Assay

Immortalized cells were seeded in a 12-well plate (HN5:100–600 cells/well, CAL27:200–1200 cells/well) and irradiated (0–6 Gy) 24 h post-seeding. Normal cell lines- fibroblast 3T3J2 (1000–4000 cells/well in 6-well plate) or oral keratinocytes HOK16B (200–800 cells/well in 12-well plate)-were also evaluated (0 or 4 Gy) 24 h post-seeding. Immediately after radiation cells were treated with palbociclib (0–1 µM). After every 72 h, media was replaced with drug-free medium and assessed for clonal expansion on day 10 (HN5) or 12 (CAL27). Plates were washed with 1× PBS; cells were fixed, stained with 0.25% Cresyl Violet for 30 min, washed with water, and allowed to air dry. Colony images were scanned using a digital scanner (Epson). Plating efficiency and surviving fractions (SF) were determined as previously described [33,34].

### 2.7. Determination of Drug-Radiation Combination Index Using Compusyn Analysis

The drug-radiation interaction was quantitated by the median effect principle and combination index (CI) method of Chou and Talalay [35]. CompuSyn allows automated statistical calculation utilizing a classic isobologram to quantify the CI [36]. Synergy was quantified with CI using clonogenic SF. CI values are interpreted as follows: CI > 1 antagonistic, CI = 1 additive, CI < 1 synergism [37].

### 2.8. Cell Cycle Analysis

After treatment, cells were washed in 1× PBS, trypsinized, collected and fixed in 70% ethanol with brief vortexing. On the day of acquisition, cells were washed twice in 1× PBS and stained with propidium iodide (PI) (Na.Spec Inc., 20 µg/mL in PBS with 2 mg DNAse free RNAse (RD Biosciences, Kenilworth, NJ, USA), proper unstained and untreated controls were maintained. Acquisition was performed using a flow cytometer (BD LSRII, BD Biosciences), and cytometric data were acquired and analyzed and quantified using FlowJo software (version 10.0.7; Tree Star, Ashland, OR, USA). The relative percent populations per cell cycle phases (G1, S and G2/M) were plotted as a histogram in the gated singlet population by using the cell cycle tool in FlowJo.

### 2.9. Senescence Associated (SA)-β-Galactosidase Staining 

Senescence-associated β-galactosidase (SA β-Gal) was assessed using a colorimetric SA β-Gal staining kit (Cat. No. 9860S, Cell Signaling Technology^®^, Beverly, MA, USA). After 72 h, cells were washed in 1× PBS, fixed, and stained with SA β-Gal solution according to the manufacturer’s protocol. SA β-Gal-positive cells were blue-stained, and imaging was performed in bright field (20×) on an epifluorescent microscope (ZEISS Axio Observer CLEM). Six random fields were acquired and positive cells were quantified. Percentage positive was recorded as relative to total number of cells per field. 

### 2.10. Western Blot Analysis 

Post-treatment, cells were harvested and immediately lysed on ice with radioimmunoprecipitation (RIPA) buffer containing protease and phosphatase inhibitors (Pierce, Thermo Fisher Scientific, Rockford, IL, USA). Protein concentrations of cell lysates were determined by DC Protein Assay (Bio-Rad Laboratories, Hercules, CA, USA), then subjected to SDS-acrylamide gel electrophoresis and transferred onto polyvinylidene fluoride (PVDF) membranes (Merck Millipore, Burlington, MA, USA) overnight at 4 °C at 35 V. Membranes were blocked in Bovine Serum Albumin (BSA) in Tris-buffered saline with 0.1% Tween 20 (TBS-T) (Thermo Fisher Scientific, Waltham, MA, USA) and probed overnight at 4 °C with primary antibodies (Supplementary Table S1). γ-H2AX measured dsDNA breaks, Ku80 evaluated non-homologous end-joining (NHEJ) response, and Rad51 was used as a marker for homologous recombination (HR) repair. Membranes were washed in TBS-T, probed for 1–2 h with fluorescent secondary antibodies, washed in TBS-T, and visualized using LI-COR Odyssey FC Imaging System (Li-Cor Biosciences, Lincoln, NE, USA). Protein levels were quantified using ImageJ software, ver. 1.52a. 

### 2.11. Transient Knockdowns-siRNA Transfections

To assess specificity of palbociclib-induced effects in OCSCC, knockdown was performed against CDK4 and CDK6 (Supplementary Table S2). Cells were seeded in 6-well plates 24 h prior to transfection. Briefly, 25 nmol of siRNAs were diluted in 100 μL of OPTI-MEMI (Invitrogen) and incubated for 5 min. 2 μL of Transfection reagent (DharmaFECT 1 transfection reagent, Dharmacon) was diluted separately in 98 μL of OPTI-MEMI and incubated for 5 min. Both tubes were combined and incubated for ~30 min. All incubations were at room temperature. Transfection was repeated after 24 h, and thereafter, RNAs were extracted and confirmed for knockdowns with quantitative PCR (qPCR) as detailed below. In parallel, colorimetric SA β-Gal staining was also performed. Senescence effects were compared to palbociclib and/or RT to confirm drug target specificity. 

### 2.12. RNA Isolation

Total RNA was extracted from cell cultures with TRI Reagent (T-9424; Sigma-Aldrich Chemie GmbH, Taufkirchen, Germany) and purified using the trizol manual isolation method as per manufacturer’s protocol. 

### 2.13. Real Time Quantitative Polymerase Chain Reaction (RT-qPCR) 

RNA was collected after 72 h treatment, quantified for its purity, and assessed for CDK4 and CDK6 expression. RNA (500 ng) was converted into cDNA using a one-step RNA-to-cDNA Kit [Applied Biosystems (Life Technologies Europe BV)]. Expression levels of CDK4 and CDK6 were quantified on Roche light cycler 96 machine with Taqman gene expression assays (Supplementary Table S3). 

### 2.14. Spheroid Models for Immortalized Lines

HN5 and CAL27 were used to create spheroids; 2D cultures were seeded at 70,000 cells/well in 6-well ultra-low attachment plate (Corning, Corning, NY, USA) in extracellular matrix suspension. Immortalized cell lines were grown in optimal StemPro hESC media (Invitrogen, Waltham, MA, USA) supplemented with 8 ng/mL bFGF (Invitrogen) suspended in 3% matrigel (Corning) matrix. Organoid cultures were maintained for 10–12 days by replenishing media supplemented with 3% matrigel every 3–4 days.

### 2.15. Establishing Patient-Derived Tumor Cell Culture via Conditional Reprogramming

Patient-derived tumor cells were cultured and established in vitro using conditional reprogramming (CR) method [15]. Briefly, tissue was minced into 5–6 mm fragments with sterile scalpel, and enzymatically digested for up to 2 h on rocking platform at 37 °C in F-medium containing 1X-collagenase, hyaluronidase, and dispase. Tumors were homogenized, centrifuged, washed, and maintained with 0.5 million irradiated feeder mouse fibroblast 3T3J2 cells in F medium at 37 °C and 5% CO_2_. Conditionally reprogrammed ‘CR’ cells were assigned cell line number during tumor harvesting. CR-06 and CR-018 are conditionally reprogrammed OCSCC CR cultures that have been previously described [38].

### 2.16. Treatment Validation in Patient-Derived Tumor Organoid Models

STR authenticated HPV(−) OCSCC CR cultures, CR-06 and CR-18, were grown in Cancer Tissue Originated Spheroid (CTOS) media or Clever’s media (DMEM/F-12 medium, 10 mM HEPES, 1× Glutamax, 10% FBS, 1× N-2 supplement (Gibco), 1× B-27 supplement (Gibco), 500 ng/mL human R-Spondin 3 (R&D Systems, Minneapolis, MN, USA), 10 ng/mL human EGF (PeproTech, Cranbury, NJ, USA), and 100 ng/mL human Noggin (PeproTech, Cranbury, NJ, USA) supplemented with 3% matrigel matrix in ultra-low attachment plate. Organoid cultures were maintained for 10–12 days by replenishing media supplemented with 3% matrigel every 3–4 days.

### 2.17. Culture Treatment of Immortalized and CR Organoid Models

Briefly, 10,000–40,000 cells/well were seeded in 3% matrigel for 24 h and treated with varying concentrations of palbociclib and/or RT. Cell numbers were seeded in successive increments with increasing radiation dose. Organoids were maintained in treated medium for 72 h and replenished with fresh matrigel-substituted medium every 3 days until termination on day 12. At termination, bright field imaging was performed (10×) and organoids size were quantified from representative images (50–100 organoids per group) using the “measure area” tool in freeware ImageJ available from the NIH website (http://rsb.info.nih.gov/ij (accessed on 10 August 2022)).

### 2.18. Trypan Blue Assay

Cell viability and total cell number were assessed in organoids. On day 12, organoids were collected from wells, washed in 1× PBS, and centrifuged at 300× *g* for 5 min. Supernatant was decanted and pellet was trypsinized with intermittent mixing to assure dissociation of 3D clusters. After ~20 min, cell counting was performed with 0.4% trypan blue under bright field (10×), and cell proliferation was evaluated.

### 2.19. Statistical Analysis

All experiments were performed three times in three independent trials. Statistical analysis was performed in GraphPad Prism 8 (GraphPad Software, version 8.3.0, San Diego, CA, USA). Average values were compared using two-tailed unpaired Student’s t-tests or rank-sum non-parametric tests. Multiple groups were compared using one-way ANOVA. All values are represented as mean ± SD/SEM or median with interquartile range. Differences were considered statistically significant if *p* values < 0.05. 

## 3. Results

### 3.1. Palbociclib Reduces Cell Proliferation and Results in Synergy When Combined with Radiation in HNSCC

**Figure 1:**
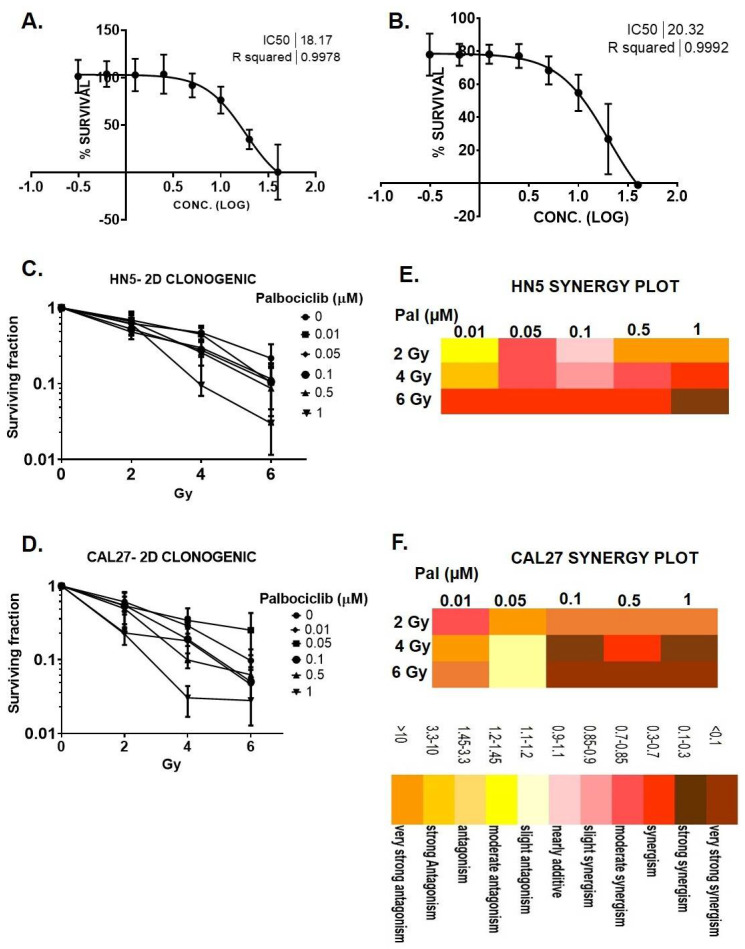
Palbociclib potentiates cell killing in HPV(−) HNSCC. HNSCC cells grown for 72 h in drug medium exhibit decreased viability in a dose dependent fashion in HN5 (**A**) and CAL27 (**B**). (**C**,**D**) Surviving fraction on day 10 in HN5 (**C**) and CAL27 (**D**) post 72 h palbociclib treatment. Synergy plot demonstrating dose response matrix for palbociclib/radiation treatment in HN5 (**E**) and CAL 27 (**F**) showed a synergistic response compared to either modality alone. Gy—Gray. Pal—palbociclib

MTT assays were performed to evaluate cellular response of HN5 and CAL27 to palbociclib. HN5 and CAL27 were significantly susceptible to palbociclib at ≥0.5 μM (Figure 1A,B). Clonogenic survival was measured after concurrent treatment of P+RT. All combinations of radiation ˃ 2 Gy and palbociclib > 0.5 μM resulted in markedly reduced SF in HN5 and CAL27 in a dose-dependent manner (Figure 1C,D). SF for combination treatment (P+RT) was decreased to 5% ± 0.05 (HN5) and 2.7% ± 0.48 (CAL27) (SF, mean ± S.D) at their highest doses compared to controls (Figure 1A,B). Interestingly, P+RT compared to palbociclib exhibited 6.7- and 2.3-fold reductions in HN5 and CAL27, respectively. Similarly, comparing P+RT to RT alone showed 1.8- (HN5) and 2.5-fold (CAL27) reductions in SF. Compusyn analysis revealed increasing synergy of the combination starting at 4 Gy and 0.5 μM doses. Although the highest synergistic effects for both HN5 and CAL27 were observed at 6 Gy and 1 μM (CI < 0.3) (Figure 1E,F), a lower radiation dose (4 Gy) was chosen for subsequent mechanistic studies.

Ideal cancer therapeutics should be effective against cancerous cells and non-toxic to normal cells. Toxicity effects of palbociclib (1 μM) were examined in HOK16B and 3T3J2 cells. We demonstrated no observable differences in cell viability in comparison to control (Supplementary Figure S1), suggesting low toxicity in normal cells. However, of note, HOK16B is HPV(+), and though HPV(+) cells are more susceptible to radiation responses, palbociclib did not enhance radiation response in HOK16B (2–4 Gy, Supplementary Figure S1A), suggesting that P+RT was differentially effective against OCSCC. In 3T3J2 cells, marginal reduction in SF was observed with palbociclib (1 μM) (Supplementary Figure S1B); however, the difference was non-significant. Effects on SF with P+RT remained similar to palbociclib alone with no resultant synergy further suggesting limited toxicity to normal cells.

### 3.2. Palbociclib Induces G1 Arrest and Potentiates Senescence When Combined with Radiation

**Figure 2:**
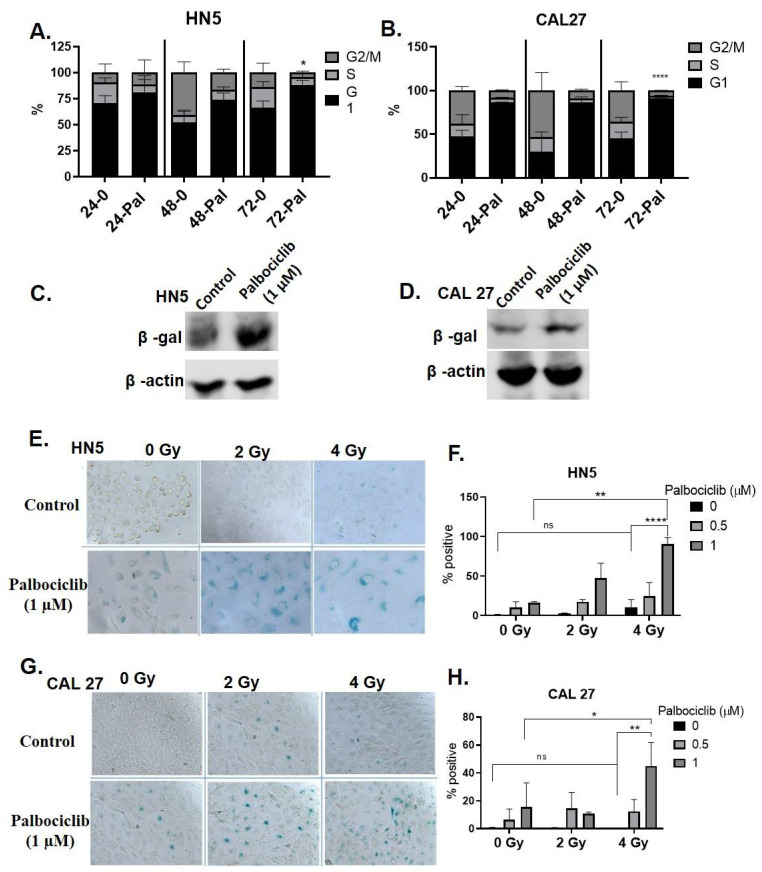
Palbociclib induces G1 arrest and senescence in HPV(−) OCSCC when combined with radiation treatment. OCSCC cells grown for 72 h in drug medium exhibited cell cycle arrest in G1 phase in HN5 (**A**) and CAL27 (**B**). The 72 h treatment resulted in senescence with SA β-gal staining using western blot and colorimetric assay in HN5 (**C**,**E**) and CAL27 (**D**,**G**). The uncropped blots are shown in Supplementary Materials. Percent β-gal-positive cell quantification for HN5 (**F**) and CAL27 (**H**). Bright field images captured at 200×. Scale: 200 µm. A 72 h palbociclib/RT (1 µM + 4 Gy) demonstrated highest levels of senescence (β-gal-positive blue-stained cells) in both HN5 (**E**,**F**) and CAL27 (**G**,**H**). Analysis by two tailed *t*-test. * *p* < 0.05; ** *p*< 0.01; **** *p* < 0.0001. Each data point represents the average percent of three individual experiments. Gy—Gray; β-gal—β-galactosidase; Pal—palbociclib.

HN5 (Figure 2A) and CAL27 (Figure 2B) exhibited a time-dependent accumulation of cells in G1 phase when treated with palbociclib from 24 to 72 h. In comparison to controls there was a 22% (*p* < 0.05) and 46% (*p* < 0.0001) increase in G1 arrested fractions in HN5 and CAL27, respectively, after 3 days of continuous treatment with palbociclib. Microscopic examination revealed flattened, irregular cells with higher cytoplasm to nuclear ratio in both the cells lines, consistent with a senescent phenotype. Palbociclib treatment caused senescence induction demonstrated with increased SA β-Gal expression in HN5 (Figure 2C) and CAL27 (Figure 2D). Time-lapse imaging validated the senescence phenotype with palbociclib after 72 h in these cells (Supplementary Videos S1 and S2). Palbociclib or P+RT treated cells indicated morphological senescent characteristics. Colorimetric SA β-Gal staining demonstrated a high percentage of cells with positive expression, again associated with enlargement and flattened phenotype in palbociclib treated cells. These phenotypic changes were more pronounced with the concurrent combination of P+RT in both HN5 and CAL27 in a dose dependent fashion (Figure 2E,G). Increases in β-Gal-positive blue cells of 16% (HN5, Figure 2F) and 15% (CAL27, Figure 2H) were observed with palbociclib compared to controls, while with P+RT (1 μM and 4 Gy), 90.4% HN5 (*p* < 0.01) (Figure 2F) and 45% CAL27 (Figure 2H) (*p* < 0.05) cells were β-Gal-positive compared to palbociclib alone. Interestingly, when radiation was used as a single modality (4 Gy), senescence was almost absent in HN5 (9%) or CAL27 (1.8%). These irradiated OCSCC’s were similar to their controls with an insignificant population of cells undergoing senescence, suggesting resistance to radiation response. Overall, P+RT significantly induced senescence in comparison to either modality alone in both the cell lines.

### 3.3. A Concurrent Palbociclib and Radiation Treatment Induces DNA Damage and Diminishes DDR Activity 

**Figure 3:**
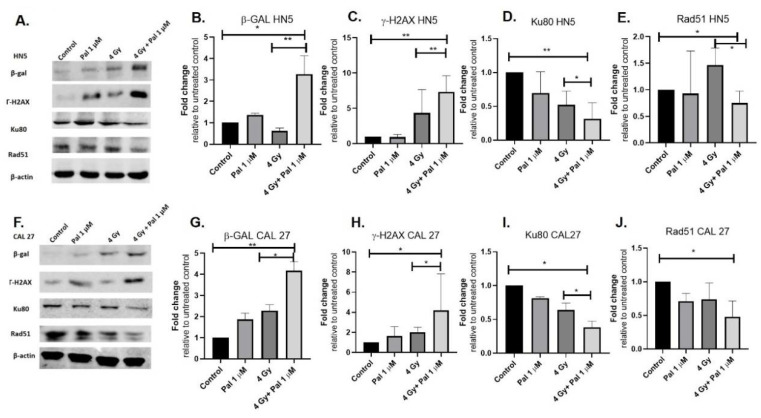
Palbociclib induces senescence in HPV (-) OCSCC and results in increased DNA damage and repression of key DNA damage repair proteins. HN5 and CAL27 western blots demonstrated increased SA β-gal expression after 72 h palbociclib treatment (**A**,**B**,**F**,**G**).The uncropped blots are shown in Supplementary Materials. Quantification of western blots for HN5 (**B**–**E**). Palbociclib/RT (4 Gy + 1 µM palbociclib) for 72 h in HN5 demonstrated increased γ-H2AX expression consistent with increased DNA damage induction (**A**,**C**); reduced expression of NHEJ signaling molecule Ku80 (**A**,**D**) and HR signaling molecule Rad51 (**A**,**E**). Quantification of western blots for CAL27 (**G**–**J**). Palbociclib/RT (4 Gy + 1 µM palbociclib) for 72 h in CAL27 demonstrated increased γ-H2AX expression consistent with increased DNA damage induction (**F**,**H**); reduced expression of NHEJ signaling molecule Ku80 (**F**,**I**) and HR signaling molecule Rad51 (**F**,**J**). Analysis by two-tailed *t*-test. * *p* < 0.05; ** *p* < 0.01. Each data point represents the average represented from three individual experiments. Pal—palbociclib; Gy—Gray; SA β-gal—senescence-associated β-galactosidase; Pal—palbociclib; HR—homologous recombination; NHEJ—non homologous end joining.

Western blots were used to demonstrate increased SA β-Gal expression in HN5 and CAL27 with P+RT compared to palbociclib or radiation alone. Palbociclib induced higher β-Gal expression compared to controls (~1.5 folds HN5 and 2 folds in CAL27), while there werethree-fold (HN5) and four-fold (CAL27) senescence increases with P+RT. P+RT potentiated senescence by 1.5 and 2-fold in HN5 and CAL27, respectively, compared to palbociclib alone (Figure 3A,B,F,G). Radiation alone did not induce significant senescence expression in HN5 or CAL27 compared to controls (Figure 3), consistent with our previous observation using colorimetric staining.

DNA damage and repair activity were assessed to provide mechanistic insight into the synergistic effects noted with P+RT in viability assays. γ-H2AX was measured 6 h after treatment. As expected, radiation did result in DNA damage, but it was not robust in either cell line, suggesting inherent mechanisms of radiation resistance in these lines. However, combining radiation with palbociclib enhanced the induced damage effects. Seven-fold and four-fold increases in the γ-H2AX expression were observed in HN5 and CAL27, respectively, with P+RT compared to either controls or palbociclib (Figure 3A,C,F,H). Key DDR proteins Ku80 and Rad51 were evaluated to examine the activity of the key DDR molecules. No significant differences were observed in Ku80 or Rad51 expression in these cell lines when palbociclib or RT was administered alone, while in combination compared to the control, P+RT showed three-fold reduction in Ku80 levels (critical for NHEJ repair) in both HN5 and CAL27 (Figure 3A,D,F,I). On the other hand, Rad51 (critical for HR repair) was repressed 1.6- (HN5) and 2.5 (CAL27)-fold with P+RT (vs. 4 Gy) (Figure 3A,E,F,J).

### 3.4. CDK4/CDK6 Knockdown Combined with Radiation Leads to Senescence and Synergism

**Figure 4:**
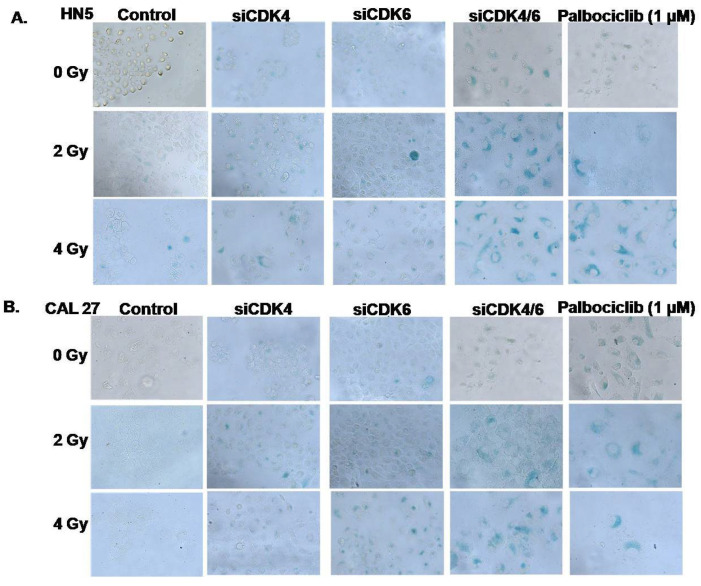
Palbociclib induces senescence in HPV(−) OCSCC, with levels recapitulated by combined knockdown of CDK4/6 and concurrent radiation treatment. OCSCC cell lines when exposed for 72 h to palbociclib exhibited senescence (SA β-gal staining) with palbociclib and radiation treatment in a dose-dependent manner. This senescence phenotype was similar to combined knockdown of both CDK4 and CDK6 together in HN5 (**A**) and CAL27 (**B**). These effects were not recapitulated with knockdown of either CDK4 or CDK6 alone. Radiation treatment at 2 or 4 Gy exhibited effects similar to respective controls. Bright field images captured at 200×. Scale: 200 µm. Gy-Gray.

To validate that CDK4/6 inhibition leads to induced senescence, transient transfection experiments were carried out in both the cell lines. The levels of CDK4 and CDK6 expression for CDK4/6 knockdowns and scramble controls were confirmed and validated with qPCR (Supplementary Figure S2A–D). Cells were also treated with palbociclib to compare the senescence response with those resulting from CDK4/6 knockdown. SA β-Gal colorimetric staining was used to evaluate and compare the resulting senescence responses under all these conditions. Transient concurrent knockdown of both the CDKs in HN5 and CAL27 showed cell flattening and enlargement similar to results noted with palbociclib treatment (Figure 4). Qualitative analysis of SA β-Gal staining was performed for all the treatments. CDK4/6 knockdown reproduced phenotypic characteristics and a SA β-Gal staining pattern similar to that seen with palbociclib treatment (1 μM) in both HN5 (Figure 4A) and CAL27 (Figure 4B). Again, radiation exposure alone did not result in a robust senescence response (Figure 2C,E and Figure 4A,B). However, a dose-dependent effect was observed combining siCDK4/6 with RT. Compared to 4 Gy RT alone, the highest combination dose (4 Gy + siCDK4/6) resulted in ubiquitous β-Gal staining in both HN5 and CAL27, demonstrating senescence (Figure 4A,B) and mirroring concurrent P+RT effects (Figure 2). Interestingly, siRNA knockdown of CDK4 or CDK6 alone, when combined with RT (Figure 4A,B), resulted in a proportionally less SA β-Gal staining compared to the combined si-RNA knockdown or compared to P+RT. These results demonstrated that downregulation of both the CDK’s was crucial to replicate palbociclib effects when combined with RT. 

### 3.5. Palbociclib Combined with Radiation Reduces Proliferation and Size of OCSCC 3D Culture Models

**Figure 5:**
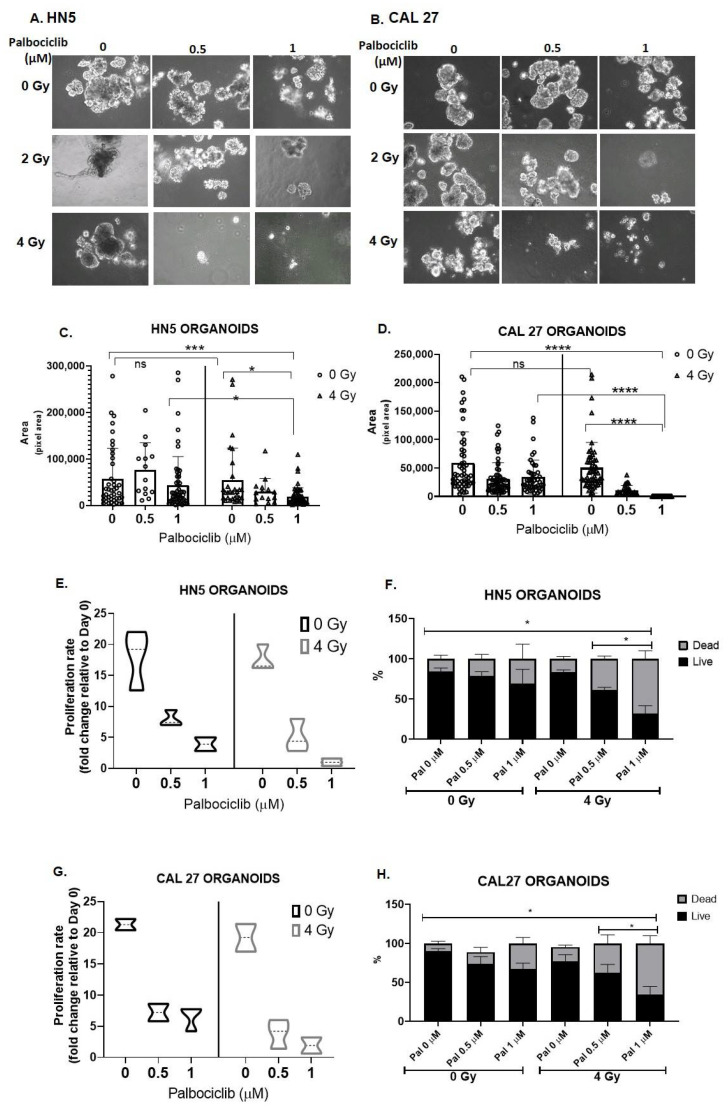
Palbociclib combined with RT results in decreased proliferation and increased cell death among HPV(−) OCSCC spheroids. HNSCC cells grown in 3D culture for 72 h in drug medium and radiation exhibits reduced spheroid formation and decreased spheroid size and viability in a dose-dependent fashion in HN5 (**A**) and CAL27 (**B**). Scale = 100 µm. Spheroid size was measured on day 12 by measuring pixel area of 50–100 random spheroids in Image J for HN5 (**C**) and CAL27 (**D**). Cell proliferation was calculated on the basis of total number of cells on the day of termination (day 12) relative to cells seeded on day 0 in HN5 (**E**) and CAL27 (**G**). Cell viability was evaluated by trypan blue assay on day 12 in HN5 (**F**) and CAL27 (**H**). The combination of palbociclib and RT demonstrated profound effects in both spheroid models. Analysis by one-way ANOVA. * *p* < 0.05; *** *p* < 0.005; **** *p* < 0.001. Each data point mean represents the average represented from three individual experiments. RT—radiation treatment; Pal—palbociclib; Gy—Gray.

Validation of cellular response to the combination treatment of P+RT in several OCSCC 3D models were examined by measuring spheroid size, rate of proliferation, and cell viability on termination (day 12). Spheroids were created using the HN5 and CAL27 immortalized cell lines. P+RT showed markedly reduced spheroid size in both the cell lines in a dose dependent manner (Figure 5A–D). CAL27 was consistently found to be more sensitive to P+RT than HN5. At the highest doses of P+RT, 2.3-fold (HN5) and 200-fold (CAL27) reductions in spheroid size were observed compared to palbociclib (Figure 5C,D), while compared to 4 Gy, there were 3-fold (HN5) and 300-fold (CAL27) reductions in the spheroid size (Figure 5C,D). Surprisingly, radiation treatment alone (vs. control) exhibited no significant reduction in size of either of these OCSCC spheroid models (Figure 5C,D). 

On termination (day 12), proliferation rates of 0 Gy and 4 Gy and their respective combination-treated cohorts were evaluated relative to the day 0 seeding. Both HN5 and CAL27 proliferated approximately 20-fold in 10 days (Figure 5E,G), consistent with a doubling time of approximately 24 h for these cell lines. Interestingly, cells irradiated alone proliferated similar to control, indicative of radiation resistance. HN5 and CAL27 exhibited proliferation rate changes of only 4- and 7-fold with palbociclib alone, while when combined with radiation (4 Gy), palbociclib further induced a greater decrease in proliferation to 2.6- (HN5) and 3.75 (CAL27)-fold (Figure 5E,G) in comparison to control. Trypan blue staining further differentiated live versus dead cells in these populations. The maximum P+RT doses tested resulted in 70% death in both the cell lines (Figure 5F,H), while palbociclib alone resulted in 30% (HN5) and 40% (CAL27) cell death, and 4 Gy alone resulted only in ~20% cell death in HN5 and CAL27. The effects of radiation on proliferation rate and cell death were found to be similar to controls, suggesting that OCSCC cell lines resist radiation responses via re-population (Figure 5C,D,F,H).

**Figure 6:**
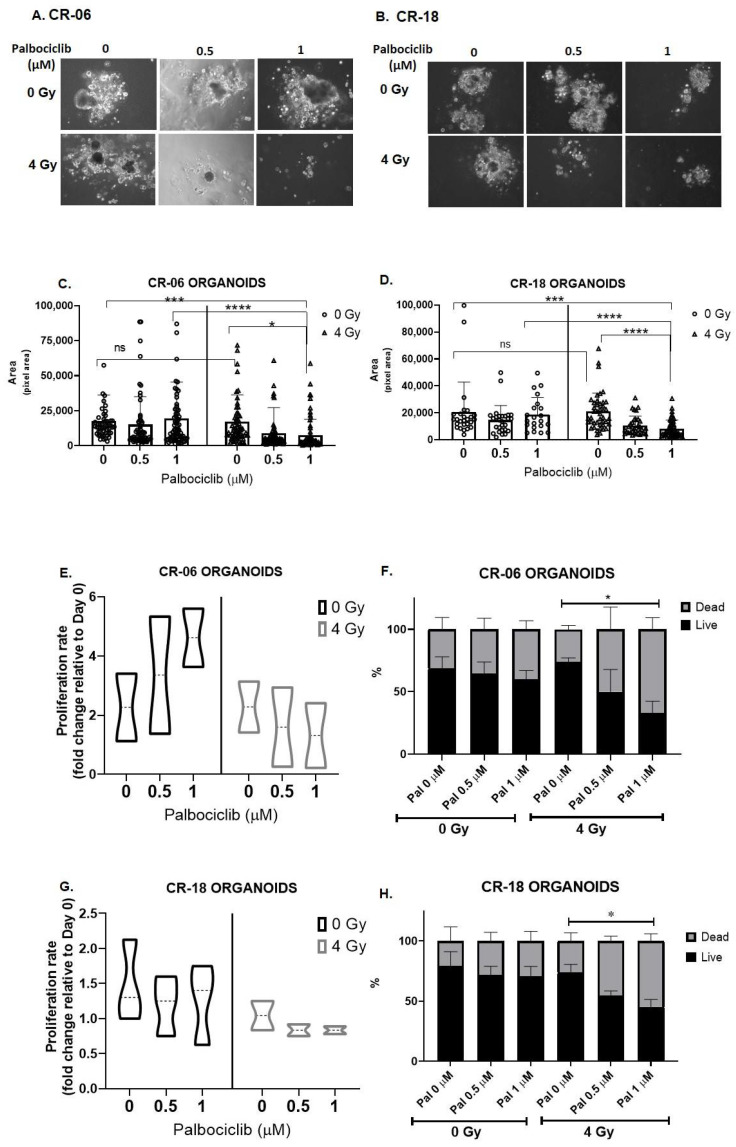
Palbociclib in combination with radiation reduces proliferation and potentiates cell killing in patient-derived HPV(−) OCSCC organoids. Patient-derived OCSCC CR cells grown in 3D culture for 72 h in the presence of palbociclib combined with radiation treatment exhibit reduced organoid formation, decreased organoid size, and decreased cell viability in a dose-dependent fashion in CR-06 (**A**) and CR-18 (**B**) Scale = 100 µm. Organoid size was measured on day 12 by measuring pixel area of 50–100 random spheroids in Image J in CR-06 (**C**) and CR-18 (**D**). Cell proliferation was calculated on the basis of total number of cells on the day of termination (day 12) relative to cells seeded on day 0 in CR-06 (**E**) and CR-18 (**G**). Cell viability was evaluated by trypan blue assay on day 12 in CR-06 (**F**) and CR-18 (**H**). Analysis by one-way ANOVA. * *p* < 0.05; *** *p* < 0.005; **** *p* < 0.001. Each data point mean represents the average represented from three individual experiments. Pal—palbociclib; Gy—Gray.

These effects were further studied in patient-derived organoids to recapitulate and validate our findings. The 3D models here are referred to as organoids as opposed to spheroids because tumor cells maintained using conditional reprogramming methods have been shown to capture heterogenous populations of cells compared to immortalized cancer cell models, which are more clonal [39]. To demonstrate that CR cultures exhibit similar responses to immortalized OCSCC cell lines, treatment of patient-derived OCSCC’s-CR-06 and CR-18 with palbociclib demonstrated senescence after 72 h (Supplementary Videos S3 and S4). These 2D culture effects were similar to HN5 and CAL27. We further ascertained the effect of P+RT combination treatment in patient-derived organoids generated from maintenance 2D CR cultures (Figure 6). Similar to immortalized cell lines (Figure 5), there was no difference in the CR organoid size between controls or cells treated with 4 Gy RT (Figure 6A–D). Additionally, palbociclib by itself did not demonstrate significant difference in the organoid sizes when compared to their respective control. However, P+RT vs. palbociclib demonstrated marked organoid size reduction: CR-06 (2.6 fold) (Figure 6C) and CR-18 (2.2 fold) (Figure 6D). On analyzing proliferation rate the total cell count relative to day 0 showed that CR-06 and CR-18 organoids proliferated at an average rate of 2.25- and 1.25-fold, respectively (Figure 6E,G). Radiation alone did not confer any significant changes in either CR-06 or CR-18 proliferation rate remaining similar to controls. Interestingly, compared to control, palbociclib alone (1 µM) enhanced the proliferation rate to 4.5 fold in CR-06 (Figure 6E), while treated CR-18 demonstrated no significant change vs. control (Figure 6G). However, at the highest treatment dose of P+RT, the proliferation rate of these patient derived organoids reduced to 1.3 (CR-06) and 0.8 (CR-18), i.e., 1.7- and 1.5-fold less than their controls, respectively.

Cellular viability was further analyzed with the trypan blue live dead staining, and 70% (CR-06) and 80% (CR-18) cells were viable in control, similar to palbociclib-treated organoid cultures (60% (CR-06) and 70% (CR 18)) (Figure 6F,H). Even though in CR-06 the total cell count was higher with palbociclib treatment, the overall viability remained unchanged, suggesting that palbociclib has no therapeutic impact as a single agent in this model, while viability with radiation alone also remained similar to the controls. Overall, palbociclib or radiation alone did not act as an effective therapeutic in the organoid models when used as single therapy. However, at the highest P+RT dose combination, the overall viability reduced to 35% (CR-06) and 40% (CR-18) (Figure 6F,H). P+RT resulted in both the lowest proliferation and largest percent dead cells.

## 4. Discussion

In contrast to HPV(−) HNSCC, HPV(+) tumors exhibit an enhanced response to radiation, and it has been hypothesized that this is at least partially due to retained function of p16 and perhaps the resulting impact on CDK4/6 signaling [10].The mechanisms by which active CDK4/6 promotes tumorigenesis and induces treatment resistance are not fully known (11). One possibility is via the suppression of senescence in response to oncogenic stress [19]. Utilizing palbociclib, an FDA-approved CDK4/6 inhibitor, on HPV(−) OCSCC cells, we observed profound senescence and increased DNA damage upon combining it with radiation, leading to synergistic cell death in both 2D OCSCC models. Synergistic effect of palbociclib was observed with radiation doses over 4 Gy. Similar treatment in fibroblasts (3T3J2) and HOK16B oral keratinocytes resulted in lack of significant response, suggesting P+RT may be toxic to OCSCC cells while sparing normal oral tissues. 

We further studied how CDK4/6 inhibition utilizing palbociclib synergized with radiation in OCSCC by impacting DDR and senescence. We know that the efficacy of cytotoxic chemotherapy and radiation are dependent on cell proliferation and cell cycle dynamics [40]. The addition of CDK4/6 inhibition to numerous established treatments in HNSCC has potential to improve responses to other therapies if these dynamics are appropriately manipulated [41]. Studies suggest palbociclib inhibits cell proliferation resulting in G1 arrest. Additionally, prolonged inhibition of CDK4/6 leads to senescence [42]. Interestingly, previous studies suggest OCSCC resists radiation treatment by *inhibiting* senescence [3,43]. Indeed, in all of our experiments, we noted little, if any, evidence of senescence in OCSCC cells after treatment with radiation alone, while we observed that OCSCC consistently demonstrated G1 arrest with palbociclib along with marked senescence (SA β-gal expression). This senescence induction was enhanced when combining palbociclib with radiation.

Palbociclib induces senescence in multiple cell types [44]. Senescence induced by RT prevents cancer cells from progressing towards malignancy [45]. Double-strand (ds)DNA breaks are most common after RT [46,47]. These (ds)DNA breaks can be repaired via NHEJ and HR. Cancer cells have the highest sensitivity to DNA damaging therapy in G2-M phase, less sensitivity in G1, and the least sensitivity in the S phase. NHEJ is active in G1, but is error-prone as it does not rely on a replicate sister chromatid [48], and hence there is some level of radiosensitivity in this stage of the cell cycle, which is exacerbated if NHEJ is impaired [49]. Meanwhile, HR is a predominant repair mechanism in the S phase through G2/M and relies on the replicate sister chromatid [50]. HR is the primary means by which cancer cells mitigate radiation damage. In our study, we observed RT caused DNA damage in HN5 and CAL27 but were not robust suggesting OCSCC resist radiation response. Our data have suggested that these lines exhibit low levels of γ-H2AX expression and minimal senescence activity after RT doses at 2, 4, and 6 Gy. Adding palbociclib to these regimens appeared to increase their sensitivity towards radiation. These findings were not only associated with increased DNA damage but also reduced expression of both NHEJ and HR related proteins and increased cell death. Collectively, these findings suggest that palbociclib significantly impairs the ability of OCSCC cells to resist radiation-induced effects.

The senescence response we observed with P+RT was consistent and profound. Senescence is a state of dormancy where DNA repair pathways are no longer available, negatively impacting both of the key DDR pathways described above [25]. We consistently observed synergy in cell death and senescence with more pronounced accumulation of DNA damage (γ-H2AX) with P+RT. These findings appeared to be driven by impaired repair mechanisms. Our observations collectively support the mechanism that palbociclib induces a predominant G1 arrest, meaning HR became largely unavailable (reduced Rad51) as a primary means of DDR. We further speculate that the senescent state disrupts the alternative NHEJ pathway, and indeed, we found that P+RT decreased Ku80 expression. Thus, we postulate, based on our observations, that the synergistic activity of combining P+RT is due to the accumulation of ds-DNA breaks and senescence secondary to the profound impact that CDK4/6 inhibition has on the ability of OCSCC cells to repair DNA damage. 

None of the earlier studies exploring combination therapy of palbociclib and radiation in HNSCC have explored this potential of simultaneous disruption of both the repair pathways. Our findings are consistent with, and expand upon, a previous study reporting that HR pathway is impaired in HNSCC after treatment with palbociclib and RT [25]. However, our study provides the first insight that combination of palbociclib and radiation impacts NHEJ also in addition to HR.

We further explored target specific activity of palbociclib in these OCSCC’s. Palbociclib is reportedly the first highly specific inhibitor of CDK4/6 with over 1,000 times greater affinity to CDK4/6 than to other CDKs [51]. Despite this, we sought to confirm that our results were specific to CDK4/6 activity, and hence, we performed transient knockdown of each kinase. Interestingly, our knockdown studies revealed that palbociclib effect is a function of both CDK4 and CDK6 inhibition as we observed increased senescence with combined inhibition compared to silencing either alone. Further, these effects increased combining transient transfection with radiation. These effects were expectedly similar to those observed with P+RT, leading to more pronounced senescence, suggesting specificity of palbociclib is towards both CDK4 and CDK6 and the combined inhibition is responsible for the induced effects. We suspect that the complex interplay of the collective impact on both CDK4 and CDK6 downstream targets are important for the profound responses that we observed, particularly those signaling cascades driving the senescence response. Our future work will focus on detailing these pathways. 

Cancer treatment is limited by inaccurate predictors of patient-specific therapeutic response; therefore, models that better predict clinical activity are needed [52]. Patient-derived tumor organoids grow rapidly and mimic tumors from which they are derived, recapitulating in vivo systems [53]. We therefore chose to evaluate our findings in 3D models derived directly from OCSCC tumors treated at our institution. Spheroids derived from the immortalized cell lines and validation of the combination therapy in patient-derived HPV(−) CR line organoid models established from oral cavity tumors revealed synergistic P+RT responses. It was interesting to note that 3D models, similar to what is observed in human tumors, demonstrated resistance to both palbociclib and single-fractions of radiation treatment. Furthermore, we were highly encouraged that combination P+RT remained effective when validated in these models. 

These results overall demonstrated how patient derived cultures, specifically the 3D models, are important for therapeutic development and could be crucial to better understand and circumvent resistance to existing therapies. Depending on the heterogonous nature of these patient-derived tumors to palbociclib, in comparison to their 2D counterpart, data clearly indicate how clinically relevant and important a head and neck cancers organoid biobank could be to improve the discovery of effective therapeutic strategies in the future.

## 5. Conclusions

Overall, our findings suggest that senescence induced by CDK4/6 inhibition in OCSCC can overcome mechanisms of resistance to RT via accumulated DNA damage due to disruption of DNA damage repair mechanisms. We speculate that most, if not all, p16-deficient HNSCC can be expected to exhibit such effects; however, an expanded evaluation is necessary. Our data supports promotion of a clinical strategy to combine CDK4/6 inhibition with radiation to treat OCSCC. 

## Data Availability

The data presented in this study are available on request from the corresponding author. Research data are stored in the lab repository and will be shared upon request to the corresponding author.

## Supplementary Materials

The following supporting information can be downloaded at: https://www.mdpi.com/article/10.3390/cancers15072005/s1, Figure S1. Palbociclib does not affect proliferation rate of normal human oral keratinocyte HOK16B or mouse fibroblast 3T3 J2 cells. HOK16B (A) and 3T3J2 (B) cells grown in 2D for 72 h in drug medium exhibited no differences in proliferation rate in comparison to either the control or the radiation. Colony formation was measured on day 12 by crystal violet staining The combination of palbociclib and RT did not demonstrate any profound effects or synergy with the concurrent dose combinations in both the models exhibiting differential effects of palbcocilib in immortalized HNSCC and normal cells. Figure S2. Palbociclib and concurrent knockdown of CDK4 and CDK6 reduces expression of CDK 4 and CDK6. HNSCC cells grown for 72 h in drug or transfection medium with the concurrent knockdown of both CDK4/6. CDK4 and CDK6 knockdown were confirmed for the cohorts by performing QPCR in HN5 (A and B respectively) and CAL 27 (C and D respectively). The treatment exhibits decreased CDK4 expression in HN5 (A) and CAL 27 (C) and CDK6 expression in HN5 (B) and CAL 27 (D). Video S1: HN5; Video S2: CAL27; Video S3: CR 06; Video S4: CR 18; Table S1: List of antibodies. Table S2: List of siRNAs. Table S3: List of gene expression assay.
