# Supplementary material for: CDK4/6 Inhibition Induces Senescence and Enhances Radiation Response by Disabling DNA Damage Repair in Oral Cavity Squamous Cell Carcinoma
Source: Cancers (Basel). 2023 Mar 28;15(7):2005. doi: 10.3390/cancers15072005 (PMC10093103; doi:10.3390/cancers15072005)
Supplement: Supplementary file 1 [file cancers-15-02005-s001.zip › Manuscript Supplementary Tables/Supplementary Table S1.pdf]

Supplementary Table S1. List of antibodies.

ANTIBODIES	Catalog No.
β galactosidase	27198S, CST
Γ-H2AX	2577, CST
Ku80	2180, CST
Rad51	SC-8349
β-actin	3700S, CST

CST: Cell Signaling Technology (Danvers, MA); SC-Santa Cruz Biotechnology, USA
