# Supplementary material for: CDK4/6 Inhibition Induces Senescence and Enhances Radiation Response by Disabling DNA Damage Repair in Oral Cavity Squamous Cell Carcinoma
Source: Cancers (Basel). 2023 Mar 28;15(7):2005. doi: 10.3390/cancers15072005 (PMC10093103; doi:10.3390/cancers15072005)
Supplement: Supplementary file 1 [file cancers-15-02005-s001.zip › Manuscript Supplementary Tables/Supplementary Table S2.pdf]

Supplementary Table S2. List of siRNAs

siGENOME HUMAN	siRNA Smart Pool Catalog No.
CDK4	M-003238-02-0005
CDK6	M-003240-02-0005
Scramble Control	D-001140-01-05
