# Supplementary material for: CDK4/6 Inhibition Induces Senescence and Enhances Radiation Response by Disabling DNA Damage Repair in Oral Cavity Squamous Cell Carcinoma
Source: Cancers (Basel). 2023 Mar 28;15(7):2005. doi: 10.3390/cancers15072005 (PMC10093103; doi:10.3390/cancers15072005)
Supplement: Supplementary file 1 [file cancers-15-02005-s001.zip › Manuscript Supplementary Tables/Supplementary Table S3.pdf]

Supplementary Table S3. List of gene expression assay.

GENE EXPRESSION ASSAYS	Catalog No.
CDK4	4331182, Hs00364847_m1
CDK6	4331182, Hs01026371_m1
GAPDH	4331182, Hs02786624_g1
