# Supplementary material for: CDK4/6 Inhibition Induces Senescence and Enhances Radiation Response by Disabling DNA Damage Repair in Oral Cavity Squamous Cell Carcinoma
Source: Cancers (Basel). 2023 Mar 28;15(7):2005. doi: 10.3390/cancers15072005 (PMC10093103; doi:10.3390/cancers15072005)
Supplement: Supplementary file 1 [file cancers-15-02005-s001.zip › Supplementary Figure S1-Final.pdf]

Supplementary Figure S1.

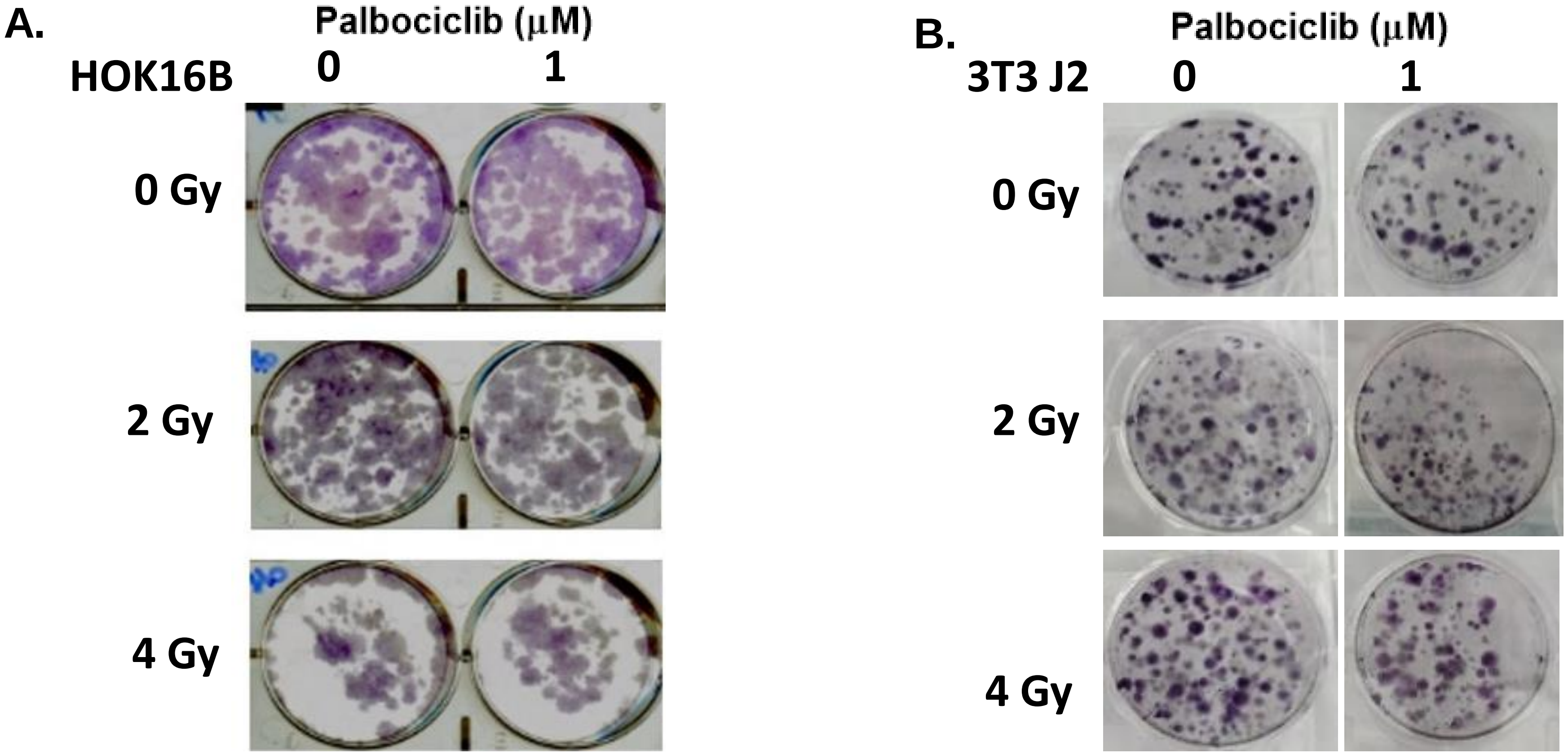

Supplementary Figure S1. Palbociclib does not affect proliferation rate of normal human oral keratinocyte HOK16B or mouse fibroblast 3T3 J2 cells. HOK16B (**A**) and 3T3J2 (**B**) cells grown in 2D for 72 h in drug medium exhibited no differences in proliferation rate in comparison to either the control or the radiation. Colony formation was measured on day 12 by crystal violet staining The combination of palbociclib and RT did not demonstrate any profound effects or synergy with the concurrent dose combinations in both the models exhibiting differential effects of palbcocilib in immortalized HNSCC and normal cells.
