# Supplementary material for: CDK4/6 Inhibition Induces Senescence and Enhances Radiation Response by Disabling DNA Damage Repair in Oral Cavity Squamous Cell Carcinoma
Source: Cancers (Basel). 2023 Mar 28;15(7):2005. doi: 10.3390/cancers15072005 (PMC10093103; doi:10.3390/cancers15072005)
Supplement: Supplementary file 1 [file cancers-15-02005-s001.zip › Supplementary Figure S2-Final.pdf]

Supplementary Figure S2.

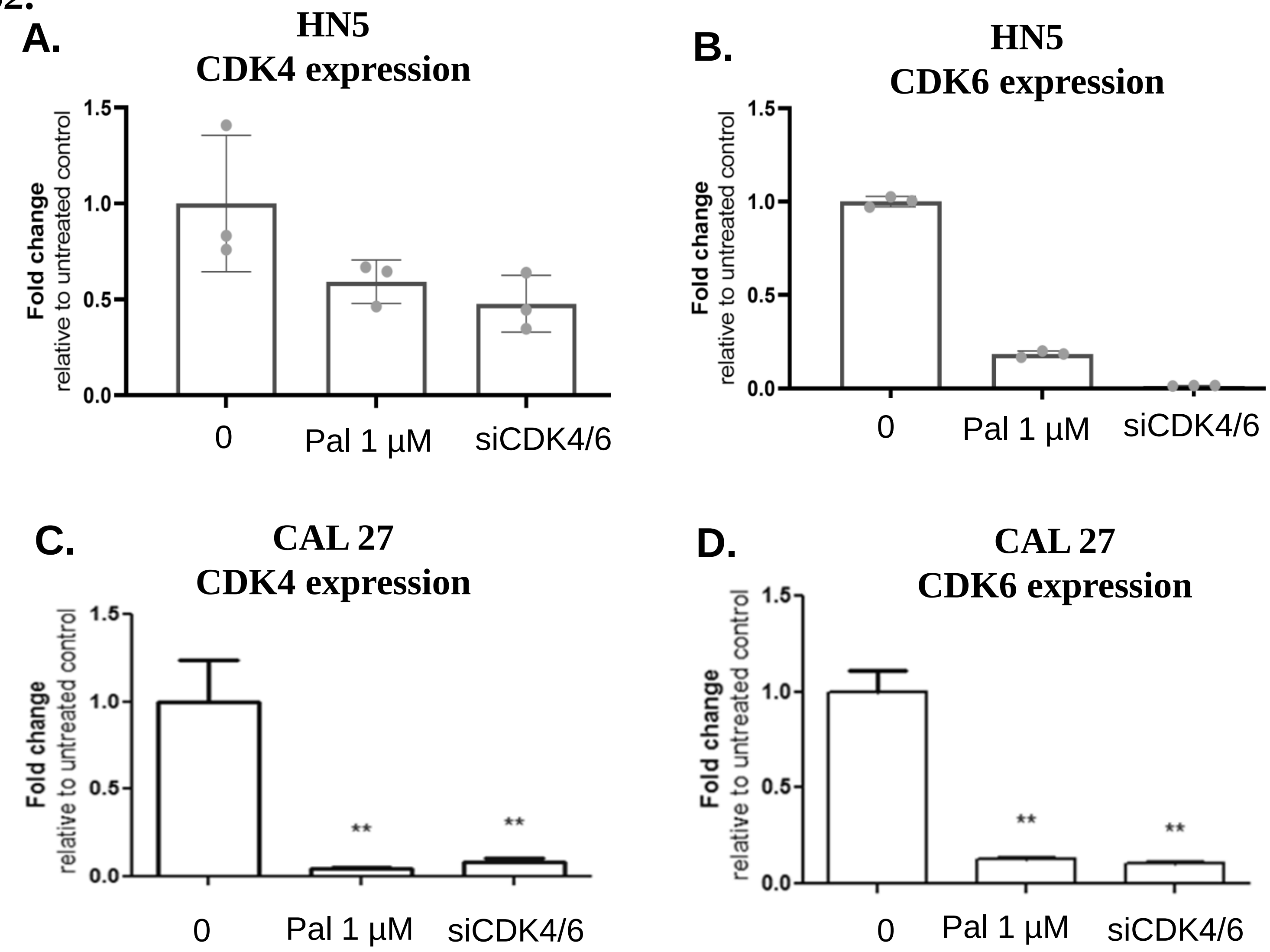

Supplementary Figure S2. Palbociclib and concurrent knockdown of CDK4 and CDK6 reduces expression of CDK 4 and CDK6. HNSCC cells grown for 72 h in drug or transfection medium with the concurrent knockdown of both CDK4/6. CDK4 and CDK6 knockdown were confirmed for the cohorts by performing QPCR in HN5 (A and B respectively) and CAL 27 (C and D respectively). The treatment exhibits decreased CDK4 expression in HN5 (**A**) and CAL 27 (**C**) and CDK6 expression in HN5 (**B**) and CAL 27 (**D**).
