# Supplementary material for: CDK4/6 Inhibition Induces Senescence and Enhances Radiation Response by Disabling DNA Damage Repair in Oral Cavity Squamous Cell Carcinoma
Source: Cancers (Basel). 2023 Mar 28;15(7):2005. doi: 10.3390/cancers15072005 (PMC10093103; doi:10.3390/cancers15072005)
Supplement: Supplementary file 1 [file cancers-15-02005-s001.zip › uncropped blots.pdf]

Supplementary supporting image

Western blot ImageJ densitometry values

Notations

0: Control
0.5: Palbociclib 0.5 µM
1: Palbociclib 1 µM

2 Gy: 2 Gray
2-0.5: 2 Gy+ Palbociclib 0.5 µM
2-1: 2 Gy+ Palbociclib 1 µM

4 Gy: 4 Gray
4-0.5: 4 Gy+ Palbociclib 0.5 µM
4-1: 4 Gy+ Palbociclib 1 µM

HN5-CAL27-β-GALACTOSIDASE (β-GAL)

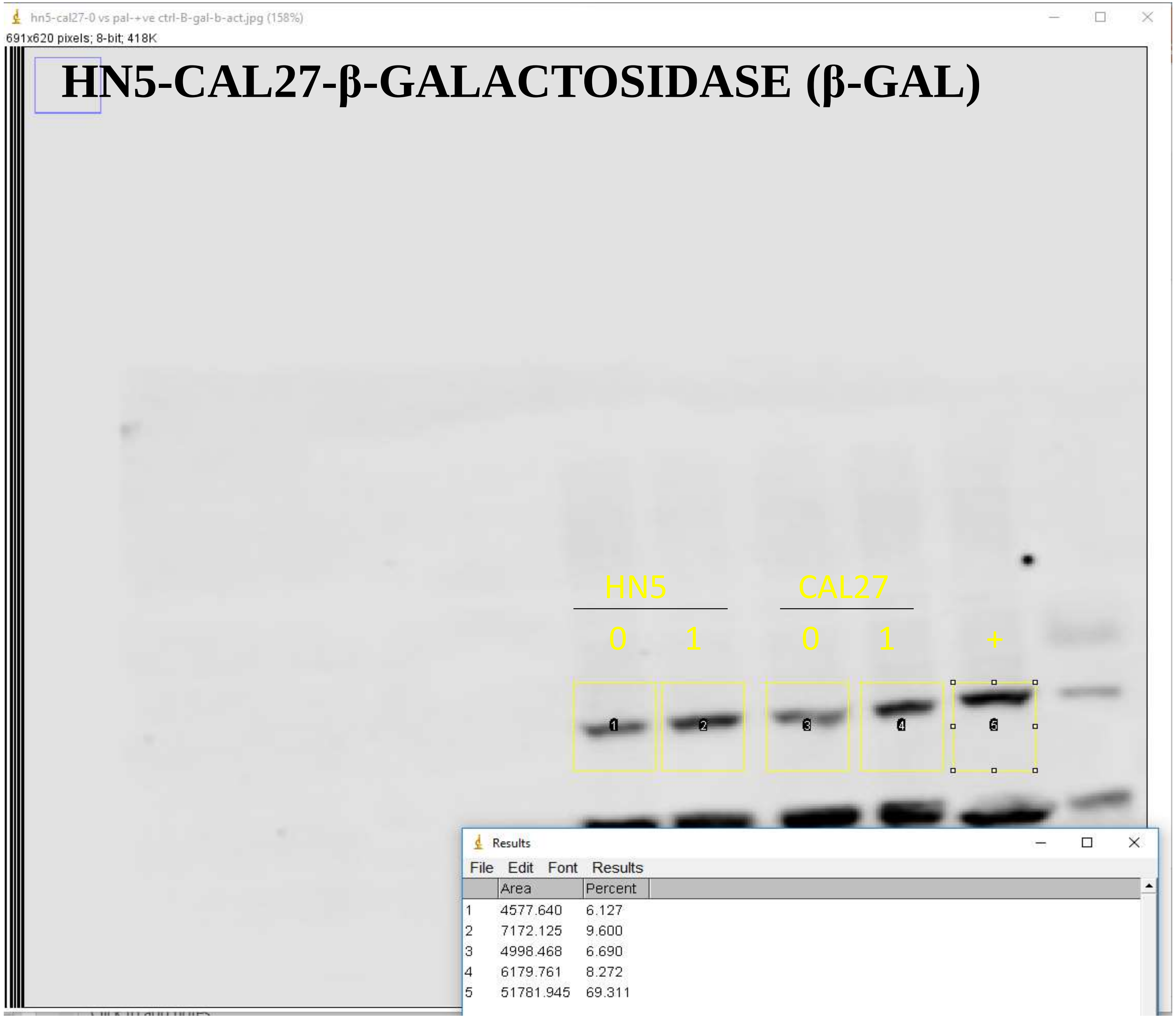

	Area	Percent
1	4577.640	6.127
2	7172.125	9.600
3	4998.468	6.690
4	6179.761	8.272
5	51781.945	69.311

HN5-CAL27-β-ACTIN

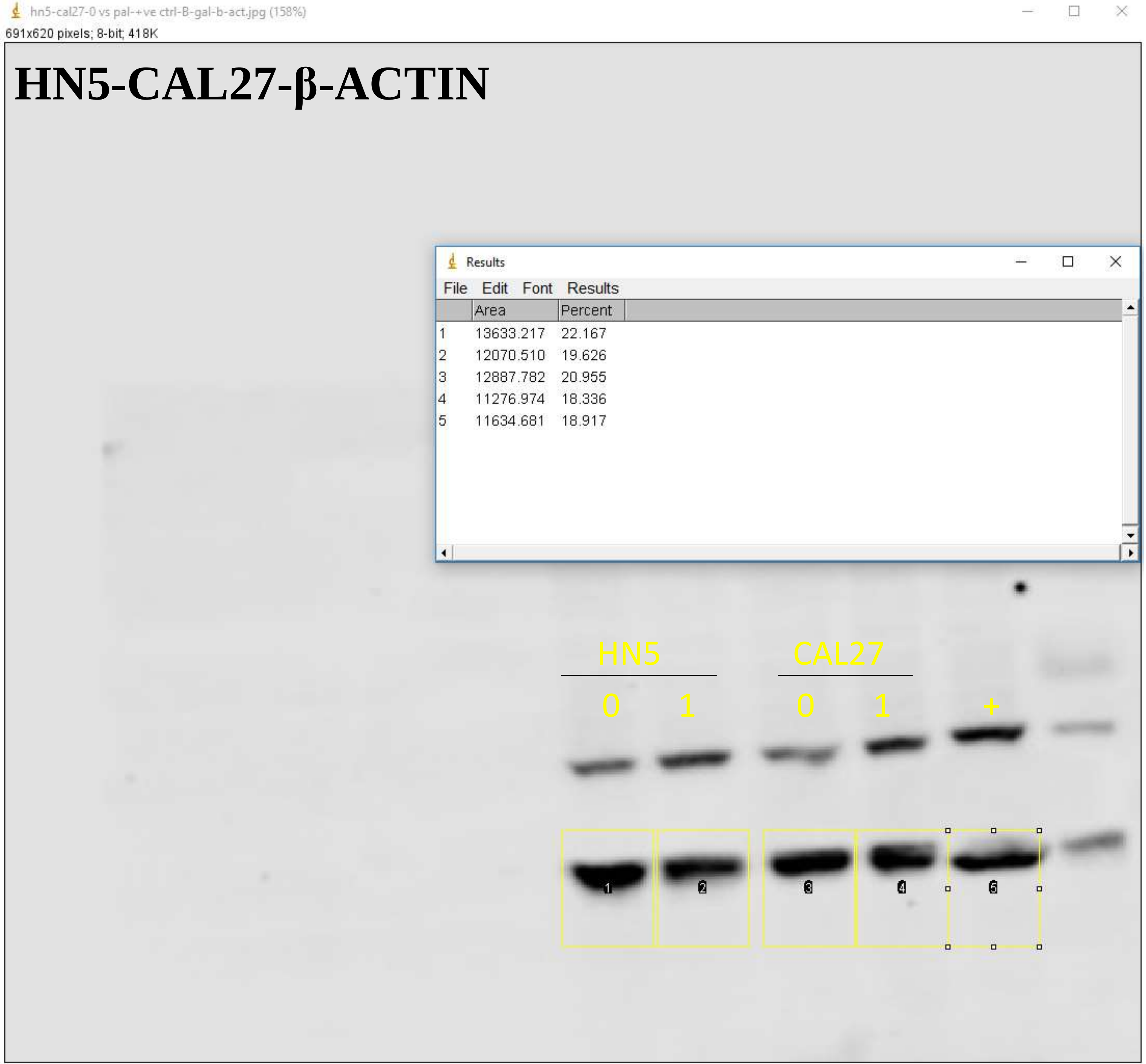

	Area	Percent
1	13633.217	22.167
2	12070.510	19.626
3	12887.782	20.955
4	11276.974	18.336
5	11634.681	18.917

hn5 rad-pal-b gal.jpg (116%)

999x845 pixels; 8-bit; 824K

HN5-β-GALACTOSIDASE (β-GAL)

Results

File Edit Font Results

	Area	Percent
1	4262.912	9.860
2	4428.962	10.244
3	6420.912	14.852
4	2139.426	4.949
5	4294.033	9.932
6	4207.962	9.733
7	3378.790	7.815
8	6247.447	14.451
9	7852.811	18.164

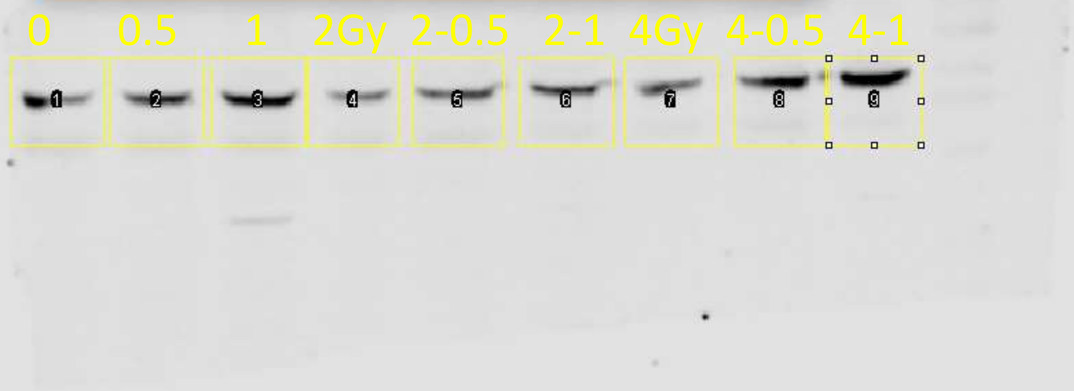

HN5-β-ACTIN

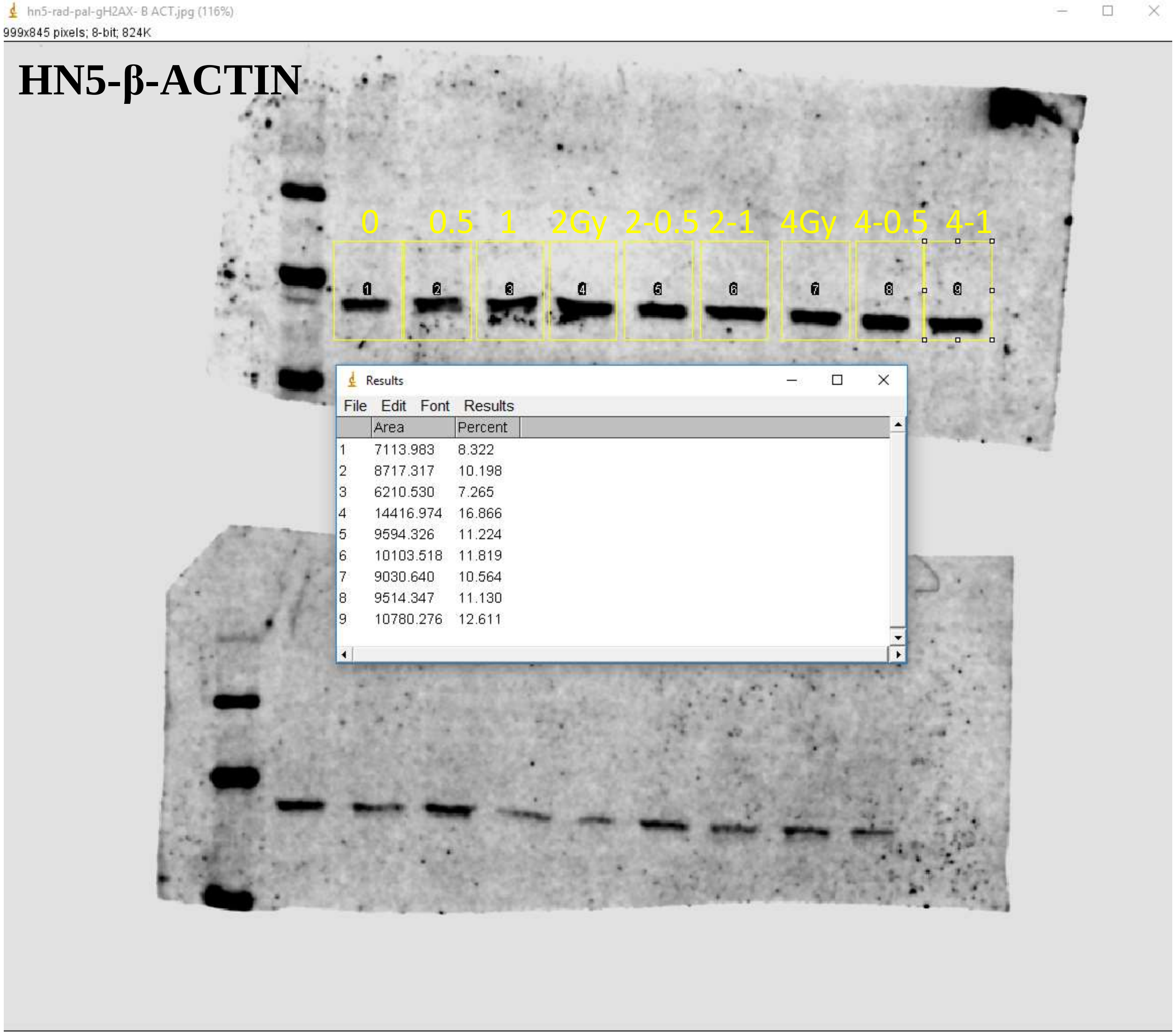

	Area	Percent
1	7113.983	8.322
2	8717.317	10.198
3	6210.530	7.265
4	14416.974	16.866
5	9594.326	11.224
6	10103.518	11.819
7	9030.640	10.564
8	9514.347	11.130
9	10780.276	12.611

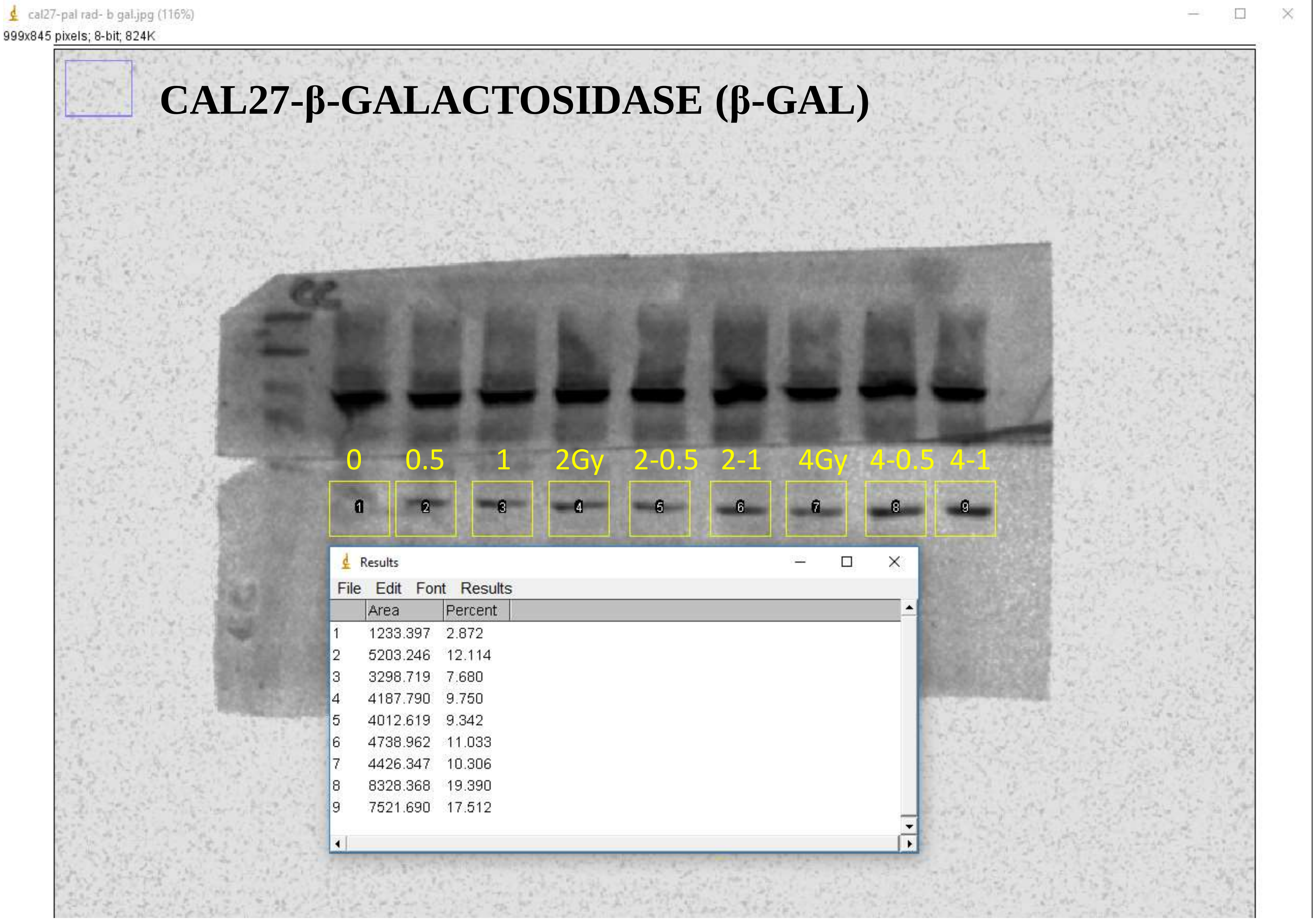

	Area	Percent
1	1233.397	2.872
2	5203.246	12.114
3	3298.719	7.680
4	4187.790	9.750
5	4012.619	9.342
6	4738.962	11.033
7	4426.347	10.306
8	8328.368	19.390
9	7521.690	17.512

CAL27-β-ACTIN

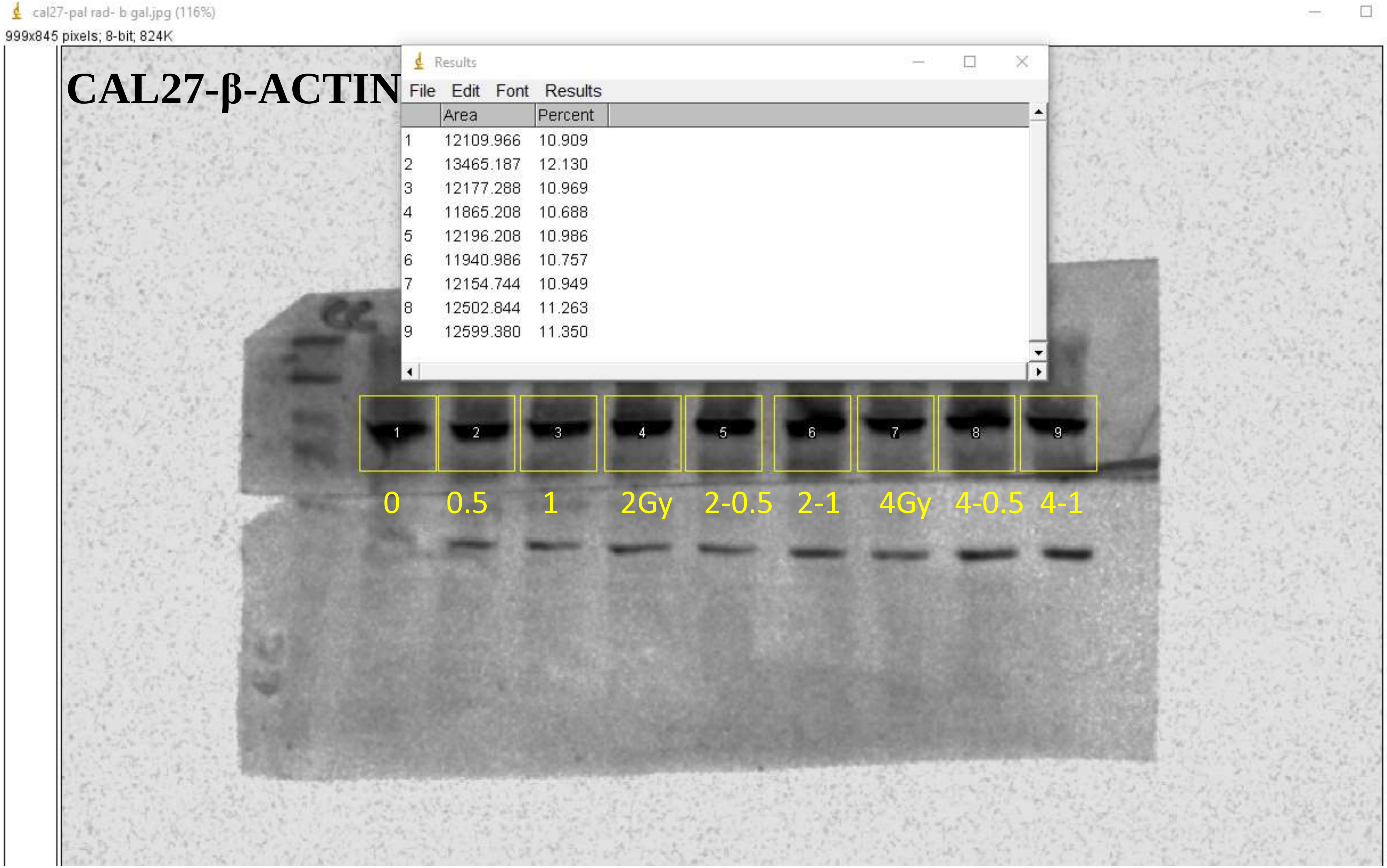

	Area	Percent
1	12109.966	10.909
2	13465.187	12.130
3	12177.288	10.969
4	11865.208	10.688
5	12196.208	10.986
6	11940.986	10.757
7	12154.744	10.949
8	12502.844	11.263
9	12599.380	11.350

CAL27-HN5-gH2AX- b act--.jpg (116%)

999x845 pixels; 8-bit; 824K

CAL27-γH2AX

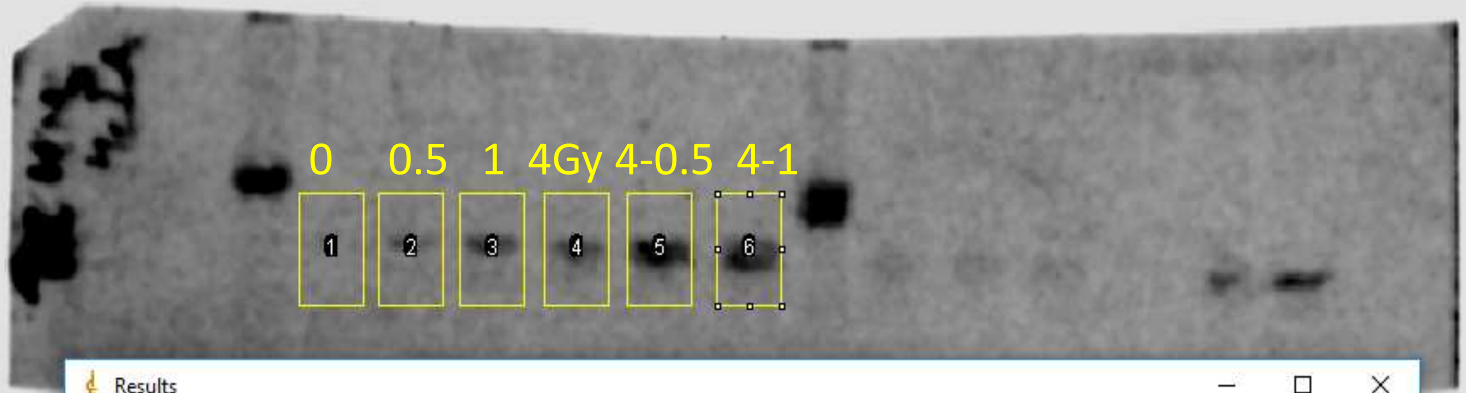

Results

File Edit Font Results

	Area	Percent
1	1078.305	2.998
2	1799.255	5.002
3	3907.397	10.862
4	5603.782	15.578
5	13128.217	36.495
6	10456.095	29.066

CAL27-β-actin

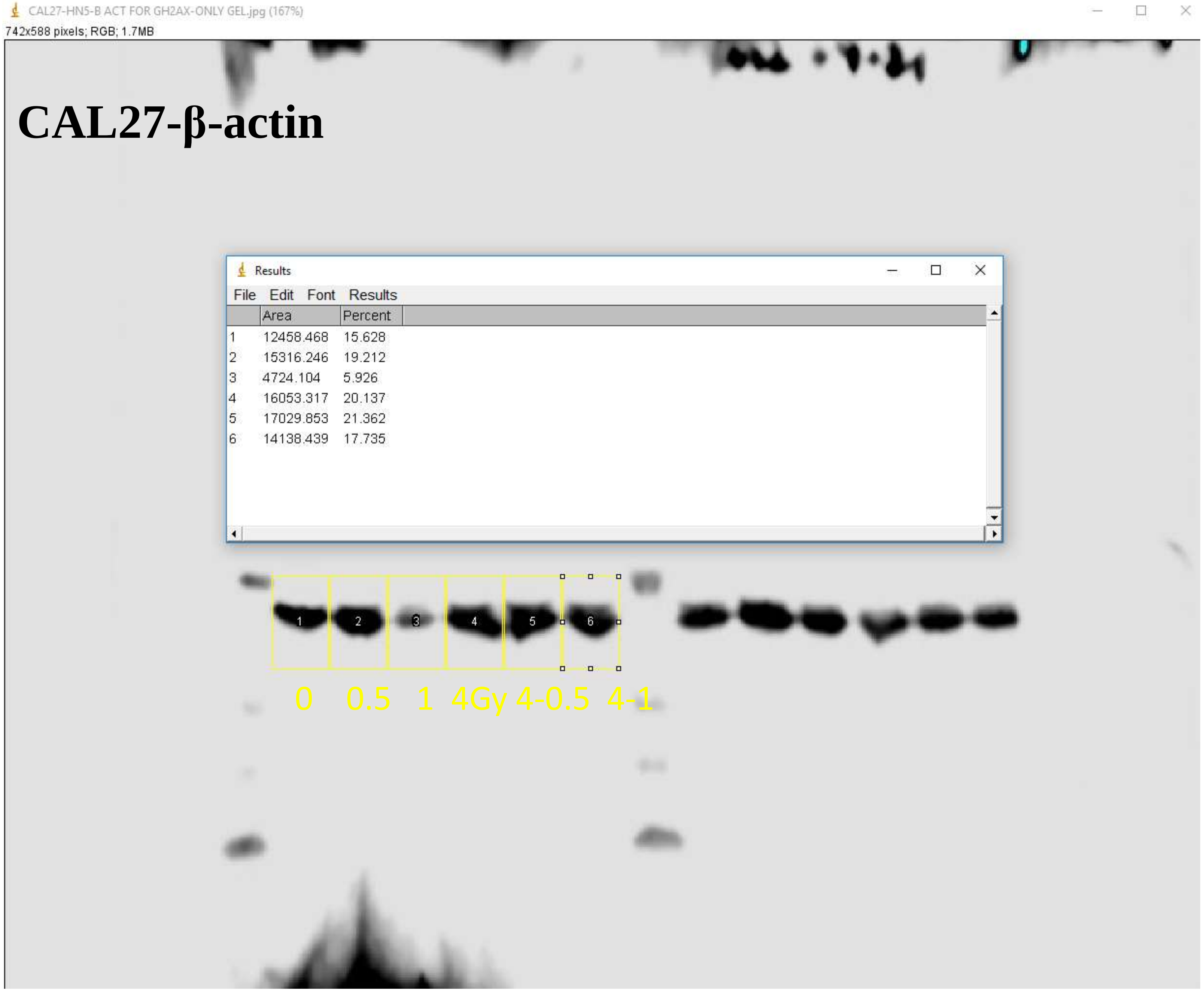

	Area	Percent
1	12458.468	15.628
2	15316.246	19.212
3	4724.104	5.926
4	16053.317	20.137
5	17029.853	21.362
6	14138.439	17.735

HN5-γH2AX

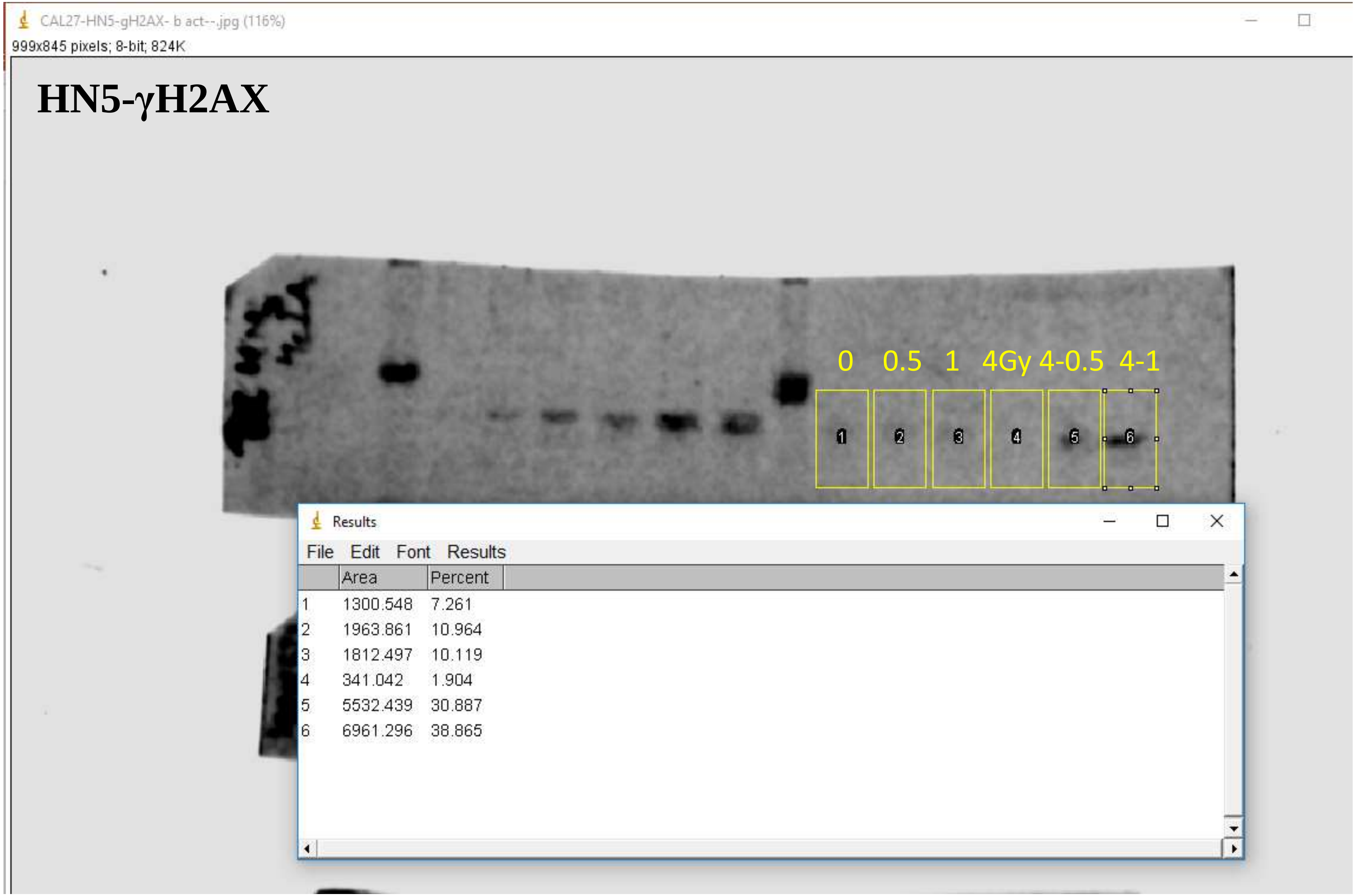

Results

File Edit Font Results

	Area	Percent
1	1300.548	7.261
2	1963.861	10.964
3	1812.497	10.119
4	341.042	1.904
5	5532.439	30.887
6	6961.296	38.865

HN5-β-actin

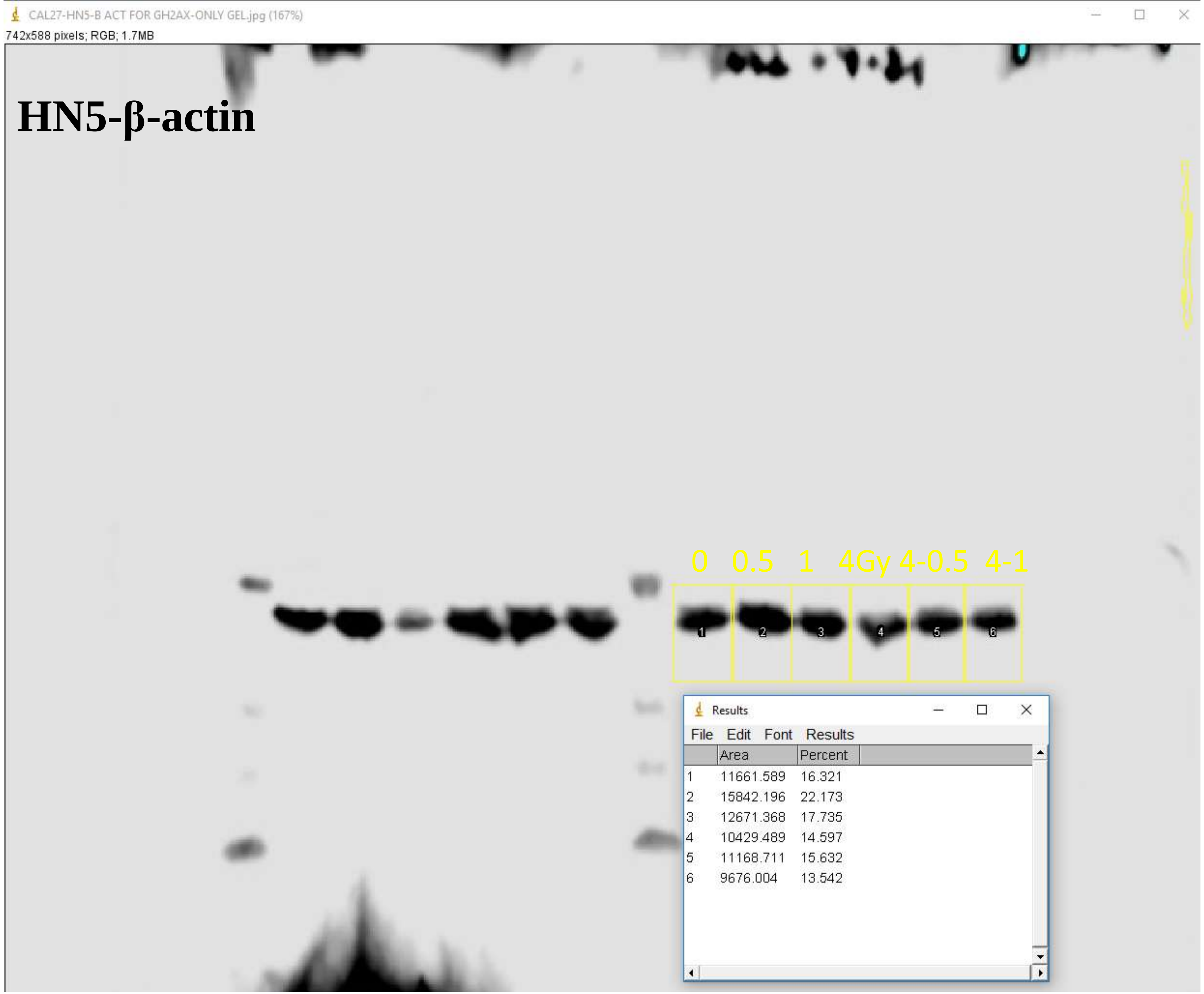
CAL27-HN5-B ACT FOR GH2AX-ONLY GEL.jpg (167%)
742x588 pixels; RGB; 1.7MB
0
0.5
1
4Gy
4-0.5
4-1
Results
File Edit Font Results

	Area	Percent
1	11661.589	16.321
2	15842.196	22.173
3	12671.368	17.735
4	10429.489	14.597
5	11168.711	15.632
6	9676.004	13.542

HN5-Ku80

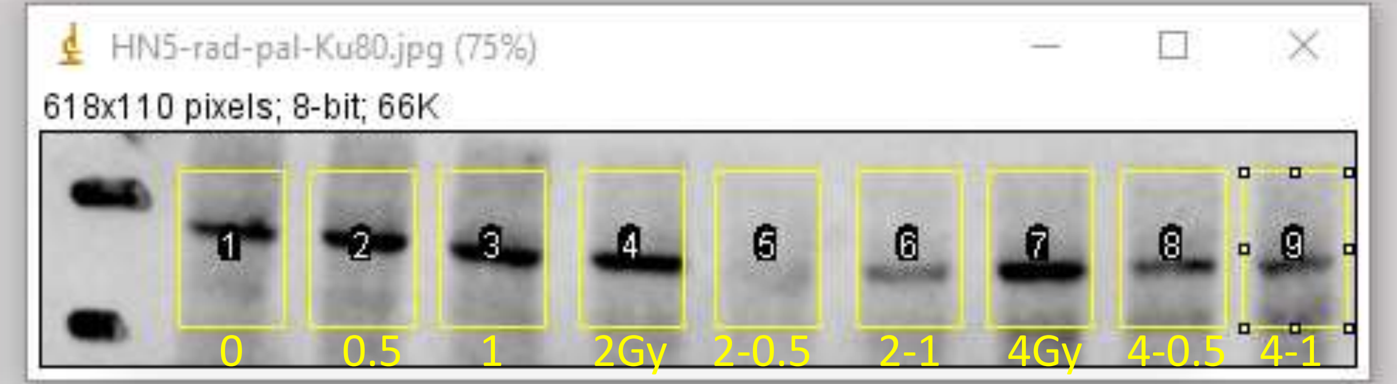

Results

File Edit Font Results

	Area	Percent
1	6522.246	12.537
2	8198.196	15.758
3	8813.439	16.940
4	8562.024	16.457
5	1132.406	2.177
6	3013.861	5.793
7	9481.995	18.226
8	3465.790	6.662
9	2835.912	5.451

HN5-β-actin

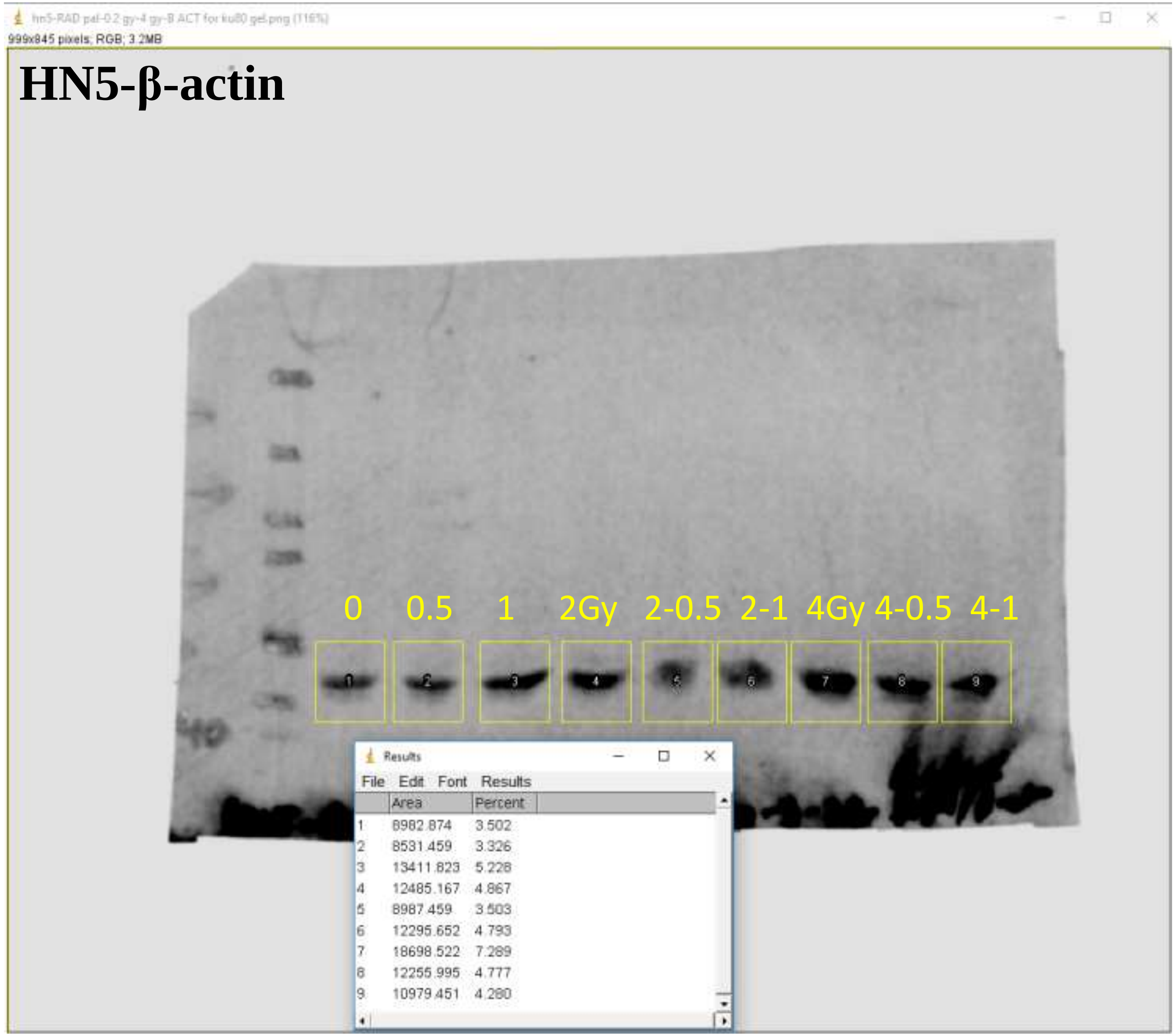

	Area	Percent
1	8982.874	3.502
2	8531.459	3.326
3	13411.823	5.228
4	12485.167	4.867
5	8987.459	3.503
6	12295.652	4.793
7	18698.522	7.289
8	12255.995	4.777
9	10979.451	4.280

CAL27-Ku80

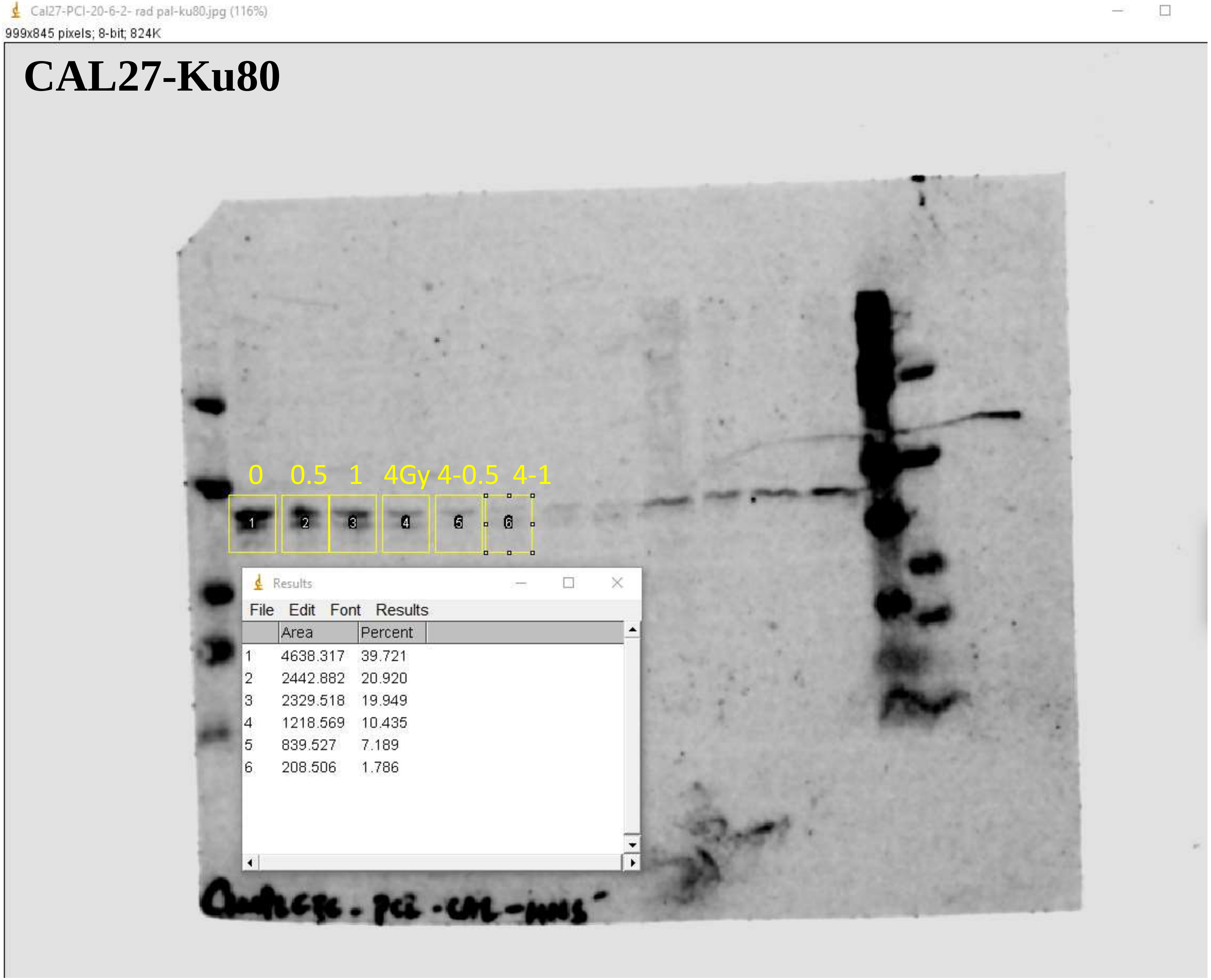
Cal27-PCI-20-6-2- rad pal-ku80.jpg (116%)
999x845 pixels; 8-bit; 824K
0 0.5 1 4Gy 4-0.5 4-1
1 2 3 4 5 6
Results
File Edit Font Results
Area Percent
1 4638.317 39.721
2 2442.882 20.920
3 2329.518 19.949
4 1218.569 10.435
5 839.527 7.189
6 208.506 1.786

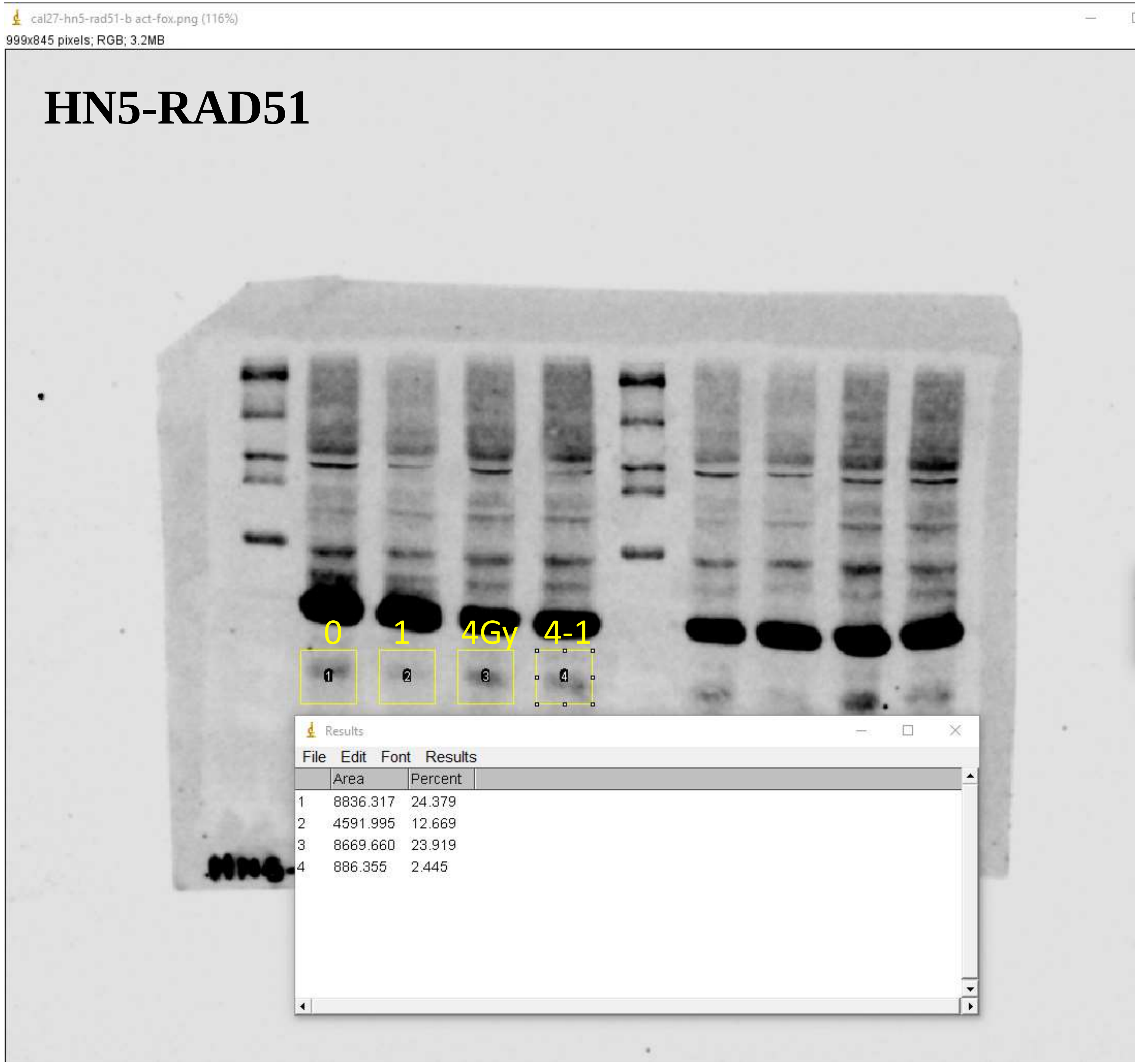

	Area	Percent
1	8836.317	24.379
2	4591.995	12.669
3	8669.660	23.919
4	886.355	2.445

HN5-β-actin

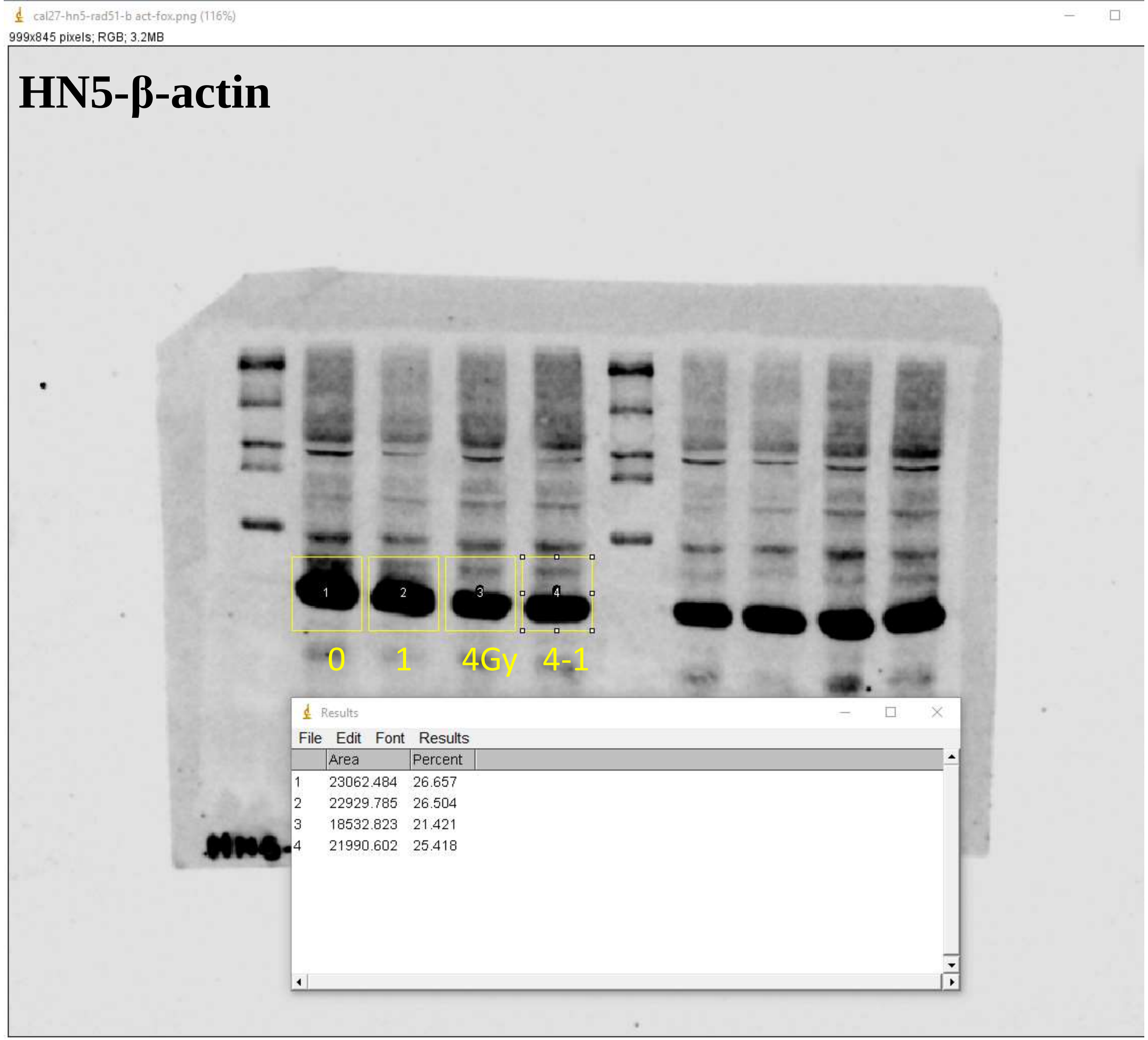

	Area	Percent
1	23062.484	26.657
2	22929.785	26.504
3	18532.823	21.421
4	21990.602	25.418

CAL27-Rad51

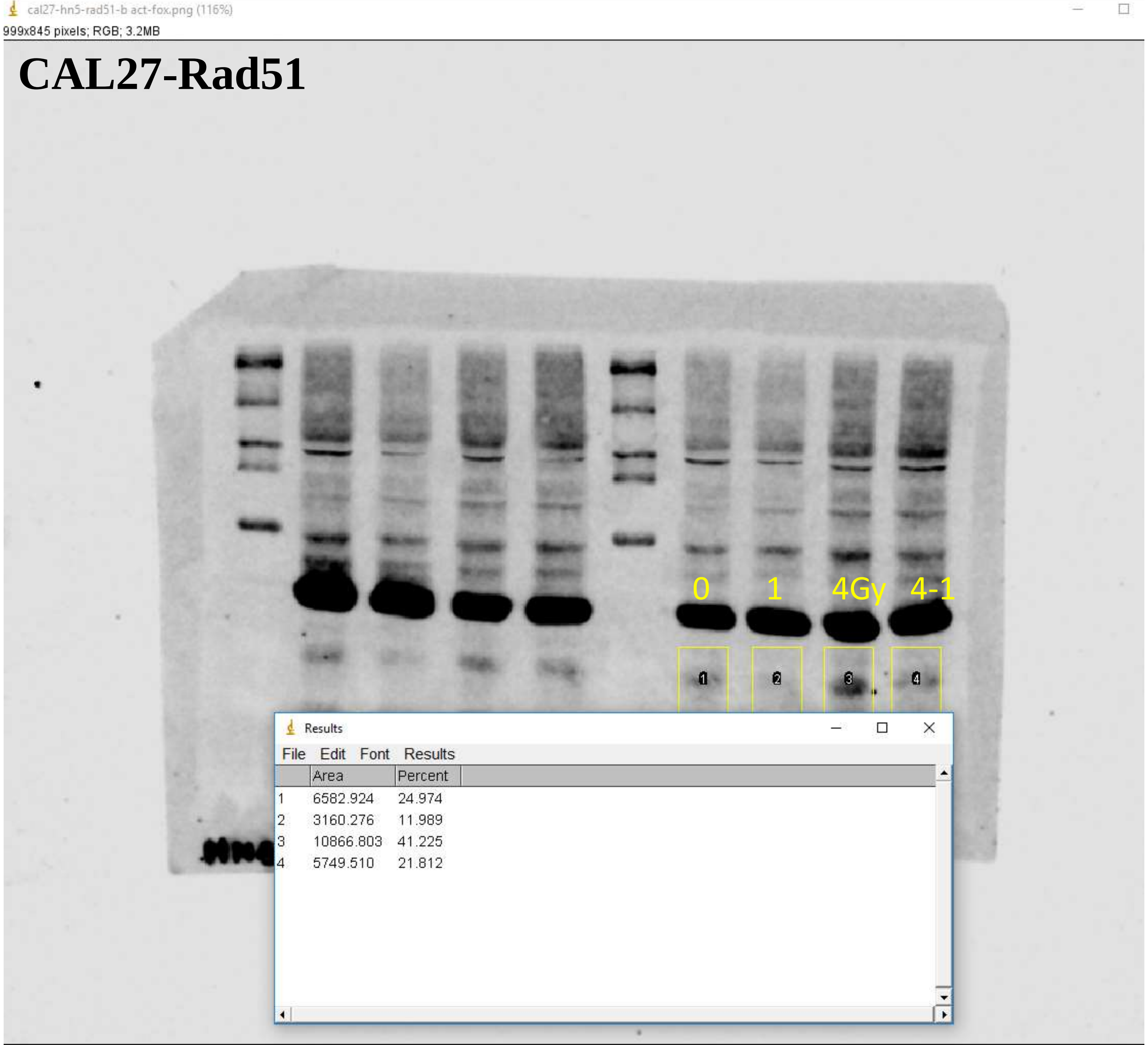

	Area	Percent
1	6582.924	24.974
2	3160.276	11.989
3	10866.803	41.225
4	5749.510	21.812

CAL27- β-actin

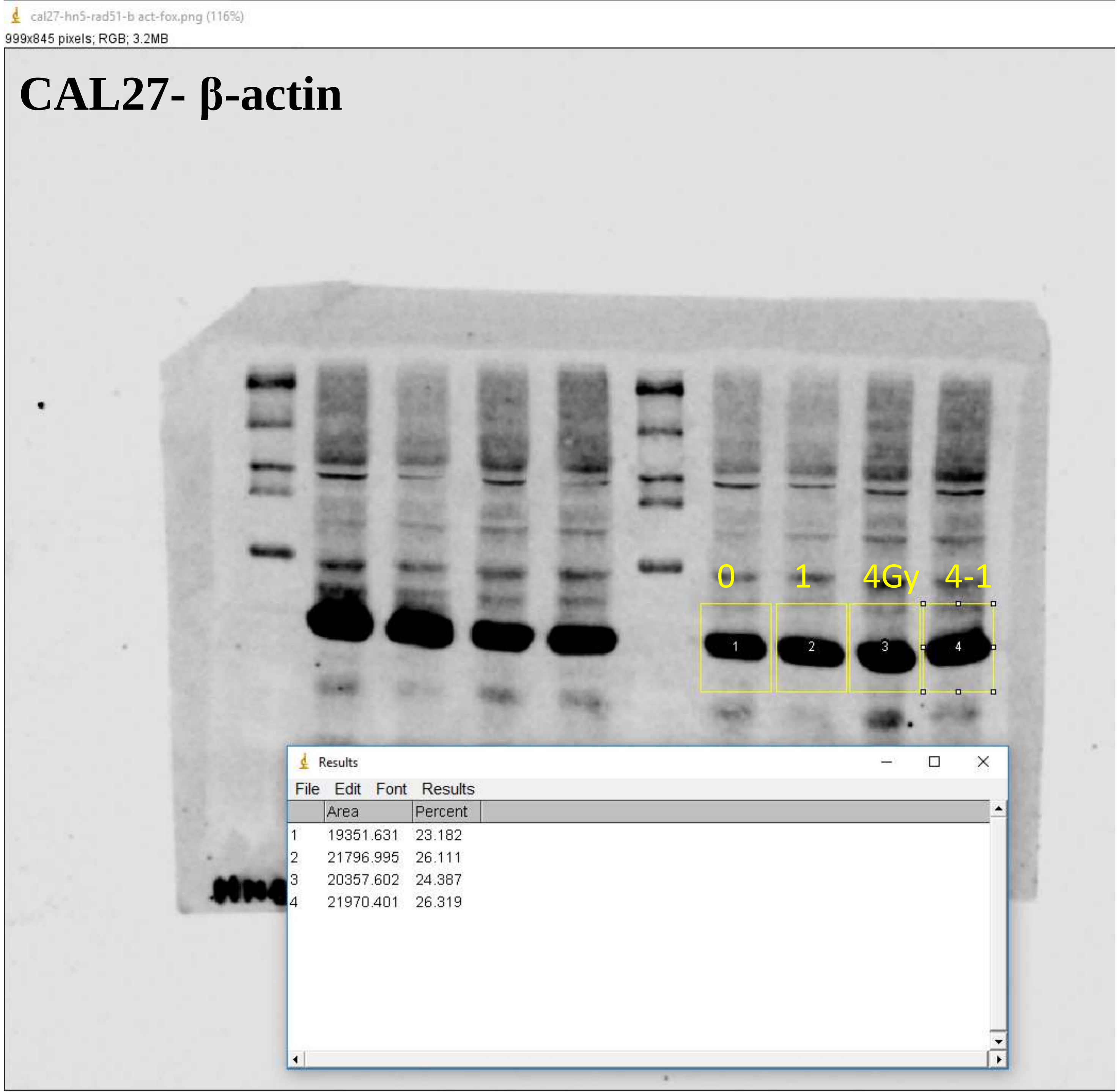

	Area	Percent
1	19351.631	23.182
2	21796.995	26.111
3	20357.602	24.387
4	21970.401	26.319
